# Auger Emitter Conjugated PARP Inhibitor for Therapy in Triple Negative Breast Cancers: A Comparative In-Vitro Study

**DOI:** 10.3390/cancers14010230

**Published:** 2022-01-04

**Authors:** Ramya Ambur Sankaranarayanan, Jennifer Peil, Andreas T. J. Vogg, Carsten Bolm, Steven Terhorst, Arno Classen, Matthias Bauwens, Jochen Maurer, Felix Mottaghy, Agnieszka Morgenroth

**Affiliations:** 1 Department of Nuclear Medicine, University Hospital Aachen, RWTH Aachen University, 52074 Aachen, Germany; rambursankar@ukaachen.de (R.A.S.); jennifer.peil@uk-koeln.de (J.P.); avogg@ukaachen.de (A.T.J.V.); matthias.bauwens@mumc.nl (M.B.); fmottaghy@ukaachen.de (F.M.); 2 Institute of Organic Chemistry, RWTH Aachen University, 52056 Aachen, Germany; Carsten.Bolm@oc.rwth-aachen.de (C.B.); steven.terhorst@rwth-aachen.de (S.T.); arno.classen@oc.rwth-aachen.de (A.C.); 3 Department of Radiology and Nuclear Medicine, Maastricht University Medical Center (MUMC+), 6229HX Maastricht, The Netherlands; 4 School of Nutrition and Translational Research in Metabolism (NUTRIM), Maastricht University, 6229HX Maastricht, The Netherlands; 5 Department of Molecular Gynecology, University Hospital Aachen, RWTH Aachen University, 52074 Aachen, Germany; jmaurer@ukaachen.de

**Keywords:** Auger emitter, TNBC, PARP1 inhibitors

## Abstract

**Simple Summary:**

Triple negative breast cancer (TNBC) is an aggressive subtype of breast cancer, with a high recurrence rate. Since treatment of BRCA^mut^ TNBC patients with PARP inhibitor (PARPi), targeting the nuclear protein PARP1, shows varied responses, its therapeutic efficacy is currently evaluated in combination with chemotherapy. Auger emitters (AEs) are radionuclides that can cause DNA damage when delivered close to the DNA. Due to the nuclear location of PARP1, radiolabelling of PARPi with AEs provide an efficient nuclear delivery mechanism. This study shows the radiosynthesis of an AE radiolabelled PARPi ([^125^I]-PARPi-01) and its therapeutic effect as monotherapy or in combination with chemotherapeutics in a panel of TNBC cell lines. We found that [^125^I]-PARPi-01 efficiently induces DNA damage with therapeutic effect irrespective of BRCA mutation. All responsive cell lines have homologous recombination deficiency. Short pretreatment with doxorubicin significantly reduces clonogenic survival of both responsive and resistant cell lines.

**Abstract:**

PARP1 inhibitors (PARPi) are currently approved for BRCA^mut^ metastatic breast cancer, but they have shown limited response in triple negative breast cancer (TNBC) patients. Combination of an Auger emitter with PARPis enables PARP inhibition and DNA strand break induction simultaneously. This will enhance cytotoxicity and additionally allow a theranostic approach. This study presents the radiosynthesis of the Auger emitter [^125^I] coupled olaparib derivative: [^125^I]-PARPi-01, and its therapeutic evaluation in a panel of TNBC cell lines. Specificity was tested by a blocking assay. DNA strand break induction was analysed by γH2AX immunofluorescence staining. Cell cycle analysis and apoptosis assays were studied using flow cytometry in TNBC cell lines (BRCA^wt/mut^). Anchorage independent growth potential was evaluated using soft agar assay. [^125^I]-PARPi-01 showed PARP1-specificity and higher cytotoxicity than olaparib in TNBC cell lines irrespective of BRCA their status. Cell lines harbouring DNA repair deficiency showed response to [^125^I]-PARPi-01 monotherapy. Combined treatment with Dox-NP further enhanced therapeutic efficiency in metastatic resistant BRCA^wt^ cell lines. The clonogenic survival was significantly reduced after treatment with [^125^I]-PARPi-01 in all TNBC lines investigated. Therapeutic efficacy was further enhanced after combined treatment with chemotherapeutics. [^125^I]-PARPi-01 is a promising radiotherapeutic agent for low radiation dosages, and mono/combined therapies of TNBC.

## 1. Introduction

Triple negative breast cancer (TNBC) is the most aggressive and heterogenous subtype of breast cancer (the most common female cancer), accounting for 15–20% of all breast cancers [1]. It is characterised by the lack of hormone receptors (progesterone and oestrogen receptors) and reduced expression of human epidermal growth factor receptor (HER2). Due to the lack of targets expressed on the surface, treatment of TNBC is confined to cytotoxic chemotherapy, immunotherapy, and/or radiotherapy.

Due to the inter- and intra-tumoral heterogeneity of TNBC, combination therapies, which overcome intrinsic and/or acquired resistance mechanisms, are favoured in comparison to monotherapies. Current FDA approved targeted therapies for TNBC include immunotherapy using the PDL1 inhibitor (PDL1i) atezolizumab (Tecentriq) in combination with nab-paclitaxel [2] or PD1 inhibitor pembrolizumab (Keytruda) combined with chemotherapy in PDL1/PD1 positive patients [3]. Additionally, the anti-Trop2 antibody-drug conjugate, sacituzumab govitecan-hziy, was approved for patients with refractory metastatic TNBC [4,5]. Patients harbouring BRCA mutation (BRCA^mut^) are treated with PARP1 inhibitors (PARPi), olaparib [6] and talazoparib [7,8,9]. However, the responses are variable, as indicated by the poor response/prognosis in several cohorts [10,11]. Hence, it is crucial to understand the responsive subtypes, and to know their molecular characterisation, for better patient selection and phenotype/molecular signature-based individualised therapy options [12].

PARP1, a nuclear protein, plays a central role as it senses several types of DNA damages, binds to the damage site, activates itself, and recruits other DNA damage repair (DDR) proteins specific to the type of repair [13]. BRCA1/2^mut^ prevalence in TNBC patients who can be receptive for PARPi therapy contribute to 10–20% of reported TNBC cases [14,15]. In the absence of BRCA1/2-mediated homologous recombination (HR), the inhibition of PARP1 blocks the activation of other PARP-1 driven DNA damage repair (DDR) mechanisms [13]. This phenomenon is called “synthetic lethality” where simultaneous loss of two compensating essential pathways cause cell death [16]. Monotherapies using PARP1 inhibitors (PARPis) target this nuclear DNA damage sensor protein PARP1. PARPis also stabilise the PARP-DNA complex, inhibiting its disassociation, thereby causing cytotoxic lesions, in turn contributing a second hit to the cytotoxicity [17]. Although PARPi monotherapy has shown efficacy in cancers with underlying BRCA1/2 mutations, it may not be efficient in HR positive or BRCA^wt^ subtypes [18]. Moreover, to avoid PARPi resistance, combination therapies are evaluated.

Combination therapies with PARPi in TNBC clinical trials predominantly focus on PARPi combined with radiotherapy, immunotherapy, or with DNA damaging chemotherapy drugs. However, treatment associated adverse effects are integral to chemotherapy and limit the tolerable dosage. Therefore, ideally maximum efficiency of DNA damage with a minimum dose should be achieved. The DNA damaging potential of internal irradiation using radioisotopes represents the most effective one. Auger electron (AE) emitters induce lethal DNA lesions with particularly high efficacy, provided it is located in close vicinity to the DNA. This is due to the high energy transferred by the released charged particles to a material, known as the linear energy transfer (LET). AEs exhibit a LET of 10–25 keV/µm and extremely short emission range (~10 nm). When proximal to DNA (<12 Å) AE induce severe DNA lesions with ~3 single strand breaks (SSBs) per decay accompanied by double strand breaks (DSB’s) [19]. Particularly, the DNA bound Auger emitter ^125^I is capable of showing high LET characteristics with SSB:DSB ratio of 6:1 [20]. Due to this unique single cell killing potential, AEs such as ^123^I, ^125^I, or ^99m^Tc have been increasingly investigated for cancer endogenous radiation therapies [21,22,23]. Apart from this therapeutic usage, their γ-emission based SPECT imaging ability can give them an advantage as a promising theranostic probe.

PARP1 is an attractive target for AE delivery proximal to DNA, because of its intranuclear localisation. Using PARPi as a vehicle provides a mechanism of AE delivery to DNA, a strategy that might avoid the need for additional usage of DNA damaging chemotherapeutic agents. In this study, we report the radiosynthesis of an Auger emitting PARPi ([^125^I]-PARPi-01) derived from the structure of the clinical PARPi olaparib. This is the first study investigating the therapeutic effects of [^125^I]-PARPi-01 as mono/combined therapies across a panel of TNBC cell lines comprising BRCA1^wt^ and BRCA^mut^ subtypes. We found that, irrespective of the BRCA status, TNBC cell lines with mutations in PI3K pathway respond to [^125^I]-PARPi-01 monotherapy at a considerably lower dosage than a pharmacological dosage used in clinic. A short pretreatment with Dox-NP (100 nM) significantly enhanced mitotic arrest in resistant metastatic cell lines and reduced the clonogenic survival.

## 2. Materials and Methods

### 2.1. Synthesis and Characterization of PARP-01 Precursor

See supporting information with NMR spectra (Figure S1A–D).

### 2.2. Radiosynthesis of [^125^I]-PARPi-01

The radionuclide ^125^I was purchased from Perkin Elmer (Boston, MA, USA). The delivered [^125^I] iodide in dilute NaOH was concentrated by evaporation and redissolution in the needed volume but did not exceed a concentration of 0.5 M NaOH. Moreover, 5 µL of a PARP-01 precursor solution (15 mM in DCM) was evaporated in a microcentrifuge tube by a N_2_ gas stream and redissolved in 20 µL of HOAc. The identity of the PARP-01 precursor was 4-{[4-fluoro-3-({4-[4-(tributylstannyl)benzoyl]-1-piperazinyl}carbonyl)phenyl]methyl}-1(2H)-phthalazinone. Moreover, 10 µL of the concentrated radioiodide solution was added to the tube, and labelling was started by adding 20 µL of a fresh chloramine-T solution (1.0 mM in MeCN). The reaction was maintained for 10 min at room temperature (24 °C) and the crude reaction mixture was purified using an analytical HPLC method (column: LiChrospher 100 RP18 EC 5 µm, 250 × 4 mm, flow 1 mL/min, gradient elution: eluent A = CH_3_CN, eluent B = 0.1% TFA aq, gradient: 2 → 8 min: 40 → 100% A, inject loop: 1 mL). The product fraction (6–8 min, 2 mL) was diluted with 10 mL of water and was solid phase extracted by a preconditioned Sep-Pak light C18 cartridge (WAT023501, Waters, Eschborn, Germany). The [^125^I]-PARP-01 was eluted using 1 mL of CH_3_CN and evaporated to dryness at 85 °C in a N_2_ gas stream. The residue was taken up in 60 µL of EtOH and diluted by isotonic saline until the EtOH concentration was below 8%.

### 2.3. Cell Culture

The cell-line panel consists of 11 cell lines: TNBC BRCA^wt^ (MDA-MB-231, MDA-MB-468, BT20, and Hs578T), TNBC BRCA^mut^ (HCC1937, HCC1395, SUM149PT, and SUM1315M02), non-TNBC BRCA^wt^ cell lines (MCF7, SKBR3) and a mammary epithelial cell line MCF10A as control. All cell lines were confirmed for the absence of mycoplasma. The cell lines HCC1937 and HCC1395 were obtained from ATCC and SUM1315M02 from BioIVT. The cell lines BT20, MDA-MB-231, MDA-MB-468, MCF7, Hs578T, MCF10A, SUM149PT, and SKBR3 were obtained from the Department for Gynaecology, University Hospital of Aachen. The cell lines MDA-MB-231, MDA-MB-468, SKBR3, BT20, and MCF7 were cultured in DMEM, supplemented with 5% FBS and 1% penicillin/streptomycin. Hs578T was cultured in DMEM supplemented with 5% FBS, 1% penicillin/streptomycin, and 10 µg/mL insulin. HCC1937 and HCC1395 were cultured in RPMI supplemented with 5% FBS and 1% penicillin/streptomycin. SUM149PT was cultured in F12(Hams), supplemented with 5% FBS, 10 mM HEPES, 1 µg/mL hydrocortisone, and 5 µg/mL insulin. SUM1315M02 was cultured in F12(Hams), supplemented with 5% FBS, 10 ng/mL EGF, 10 mM HEPES, 1 µg/mL hydrocortisone, and 5 µg/mL insulin. MCF10A, a human mammary epithelial cell line was cultured in DMEM:F12, supplemented with 5% horse serum, 20 ng/mL EGF, 0.5 mg/mL hydrocortisone, 100 ng/mL cholera toxin, 10 µg/mL insulin, and 1% penicillin/streptomycin. All cell lines were maintained in their culture conditions at 37 °C at 5% CO_2_ during culture and experiments.

### 2.4. Treatment Strategies

Different co-treatments or pre-treatment strategies were followed for each combined therapy. For liposomal doxorubicin and olaparib treatment, the cells were pre-treated for 6 h with 100 nM Dox-NP (Avanti^®^, Alabaster, AL, USA), followed by treatment for 60 h with 1 μM olaparib. For temozolomide (TMZ) (Sigma Aldrich, Taufkirchen, Germany) and olaparib co-treatment, the cells were pre-treated for 48 h with 50 μM temozolomide before co-treatment with 1 μM olaparib for 24 h. Single treatments with [^125^I]-PARPi-01 (~1 MBq/10^6^ cells), olaparib (1 μM), dinaciclib (5 μM; Sigma), and talazoparib (10 nM; Axon Medchem, Groningen, Netherlands.) were done for 72 h, dinaciclib (5 μM), and 1 μM olaparib co-treatment was done for 72 h. For Dox-NP and [^125^I]-PARPi-01 combination, the cells were pre-treated with 100 nM Dox-NP 6 h prior to incubation with [^125^I]-PARPi-01 (~1MBq/10^6^ cells) for 72 h. Untreated cells were incubated in growth medium supplemented with DMSO (≤0.2%) for 72 h.

### 2.5. Subcellular Fractionation

Fractionation was performed using a Subcellular Fractionation kit (78840, Thermo Fisher, Rockford, IL, USA). Cells were collected and washed after treatment; they were incubated with cytoplasmic, membrane, and nuclear extraction buffers, subsequent to one another; the fractions were collected, and protein measured using a BCA assay. Western blot was performed using the fractions (30 µg/lane) and, subsequently, the radioactivity in the blot was detected via a phosphorimager by incubating the blot with a Fujifilm imaging plate overnight followed by detection using Typhoon FLA.

### 2.6. SDS/Western Blot

SDS-PAGE was carried out using premade Any KD Mini-Protean TGX gels at 90 V for 1.5 h. After adequate protein separation, the proteins were transferred to PVDF membranes using a wet blotting (60 V, 2 h) technique. The blots were then blocked with 1% Milk/TBST and incubated with a primary antibody (anti-PARP1 (1:1000); Abcam ab227244; anti-GAPDH (1:4000); cell signalling 14C10). For the phosphorimager analysis, after blotting, the blots were sandwiched with a Fujifilm Imaging plate for Bioanalyzer (BAS-MS 2040), and incubated overnight, and the protein bound activity was imaged using a phosphorimager (Typhoon FLA 7000 version 1.2).

### 2.7. Cell Uptake Assay

1 × 10^5^ cells were seeded in 12-well plates and allowed to adhere overnight, and were treated with different mono/combined drugs, as described above. After treatment, the media was removed and the cells were incubated with [^125^I]-PARPi-01 (~50 KBq/well) in serum free media for 1, 4, and 24 h. Subsequently, the cells were washed, trypsinised, and collected. The cell-incorporated activity was detected using a Wizard^2^ Gamma Counter (PerkinElmer, Boston, MA, USA). The cells were then lysed and the protein concentration was measured by a standard Bradford assay to calculate the uptake per mg of protein. The experiments were independently carried out thrice.

### 2.8. Flow Cytometry

Annexin V apoptosis assay was performed using the Roche Annexin-V-FLUOS staining Kit. Cells were collected after treatment and pelleted by centrifugation. After a PBS wash, the cells were resuspended in Annexin-V-FLUOS labelling solution with Annexin V-FITC and propidium iodide, and incubated in room temperature for 10 min. The cells were then analysed using flow cytometry (CytoFLEX, Beckman Coulter, Krefeld, Germany). Similarly, Nicoletti assay for cell cycle analysis was performed by permeabilising cells with a 50:50 mixture of 1% sodium citrate and 1% Triton X. Subsequently the cells were supplemented with 100 ng/mL of propidium iodide and RNase A, incubated overnight, followed by flow cytometry and analysis (CytoFLEX, Beckman Coulter).

### 2.9. Microscopy

1 × 10^5^ cells were seeded on glass cover slips prior to treatment in a 24-well plate. After treatment, cells were fixed with 4% paraformaldehyde for 20 min. They were subsequently washed with PBS and blocked with 5% FBS/PBS with 0.3% Triton-X. The cells were incubated overnight at 4 °C with primary antibody (Mouse Anti-PARP1, 1:1000 (Sigma); Rabbit Anti-pH2AX, 1:500 (Cell signalling)) dissolved in blocking solution. After washing with PBS, the cells were incubated for 1 h with secondary antibodies (Goat Anti-Mouse DyLight 488, 1:2000 and Goat Anti-Rabbit Alexa Fluor 568, 1:1000) at room temperature. After the PBS wash steps, the cells were mounted with Roti Mount^®^ Vectashield containing DAPI and analysed using fluorescence microscopy (Zen Lite, Carl Zeiss, Jena, Germany). Quantification was performed using CellProfiler 3.0 software.

### 2.10. Colony Formation Assay

A soft agar colony formation assay was done to investigate the colony formation of cells post-treatment. The cells were trypsinised, collected after treatment, and counted using a haemocytometer. Moreover, 2500 cells/well were seeded in 0.3% soft agar (Sigma Aldrich, Taufkirchen, Germany) in media on a 6-well plate, over a 0.5% soft agar underlay. They were grown for about 21–22 days before staining with 4-NBT (1 mg/mL, 37 °C, overnight) and colony counting under a microscope.

### 2.11. Statistical Analysis

Data were analysed using GraphPad Prism 9 version and statistical analyses were performed using ANOVA with appropriate post-test algorithms.

## 3. Results

### 3.1. [^125^I]-PARPi-01 Is Specific to PARP1

**Figure 1:**
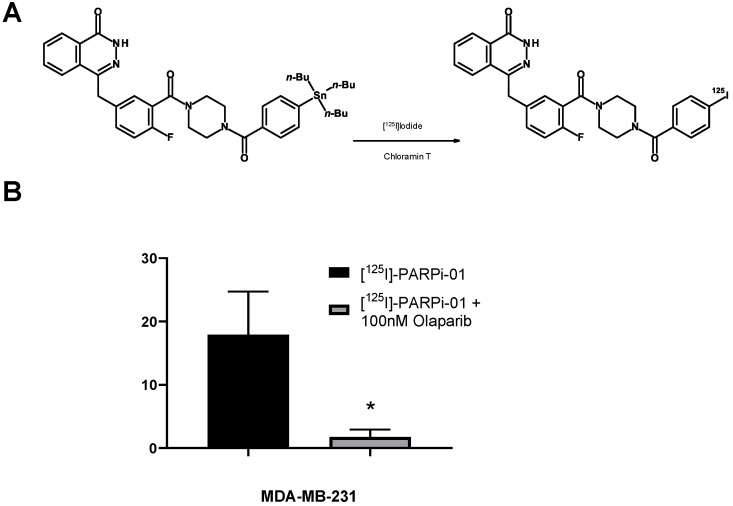
(**A**). Reaction scheme for radiolabelling with ^125^I in the presence of an oxidant to a precursor (olaparib derivative) for obtaining [^125^I]-PARPi-01. (**B**). Blocking experiment: Co-treatment of [^125^I]-PARPi-01 (1 MBq/10^6^ cells; 1 nM) with/without 100 nM olaparib and uptake measured by gamma counter 1 h post incubation. Statistical significance was tested using *t*-test (n = 3) (* *p* < 0.05).

[^125^I]-PARPi-01 was synthesised by a straightforward ^125^I-iododestannylation of tributylstannyl-benzoylated derivative of olaparib, using chloramine T as an oxidant (Figure 1A). The radiochemical purity of the synthesis was >95% with yield of 78 ± 11.8% and molar activity (MA) of 30.52 ± 6.4 GBq/µmol (n = 4) (Figure S2A). No deiodination was observed upon long-term storage at −20 °C in ethanol (Figure S2B). During the course of this study, Wilson et al. reported a similar facile approach for the synthesis of the structurally similar Auger emitting theranostic [^123^I]-MAPi, but with a significantly lower yield (45 ± 2%) and low molar activity (11.8 ± 4.8 GBq/µmol) [24]. The target specificity of the synthesised [^125^I]-PARPi-01 was evaluated in vitro (in MDA-MB-231 cells) by a blocking assay upon co-treatment of [^125^I]-PARPi-01 (1 nM) with 100× higher molar concentration of olaparib (100 nM). One-hour post incubation, co-treatment with olaparib reduced uptake (%) significantly (*p* < 0.05) to 1.15 ± 4.8% as opposed to control uptake (14.5 ± 4.8%) thereby proving target specificity (Figure 1B).

### 3.2. Breast Cancer Cell Lines but Not Mammary Epithelial Cell Line Show Retention of [^125^I]-PARPi-01

To determine therapeutic effects of [^125^I]-PARPi-01 treatment in vitro in triple negative breast cancer (TNBC), a panel of cell lines with BRCA deficient (mutant) or proficient (wild type) genotypes was chosen. Amongst the BRCA^wt^ panel, two TNBC cell lines representing the basal A subtype (BT20, MDA-MB-468) and two cell lines belonging to the aggressive basal B subtype (MDA-MB-231, Hs578T) were chosen. The panel of TNBC BRCA^mut^ cell lines consisted of representative cells from Basal A (HCC1937) and B subtypes (HCC1395, SUM149PT, SUM1315M02). Additionally, two hormone receptor positive breast cancer cell lines (MCF7, SKBR3) were included to evaluate their responsiveness, along with a non-malignant mammary breast epithelial cell line (MCF10A). In western blot analysis, TNBC cell lines did not display statistically significant differences in cellular PARP1 expression, whereas benign breast epithelial cells showed enhanced expression (Figure S3A).

**Figure 2:**
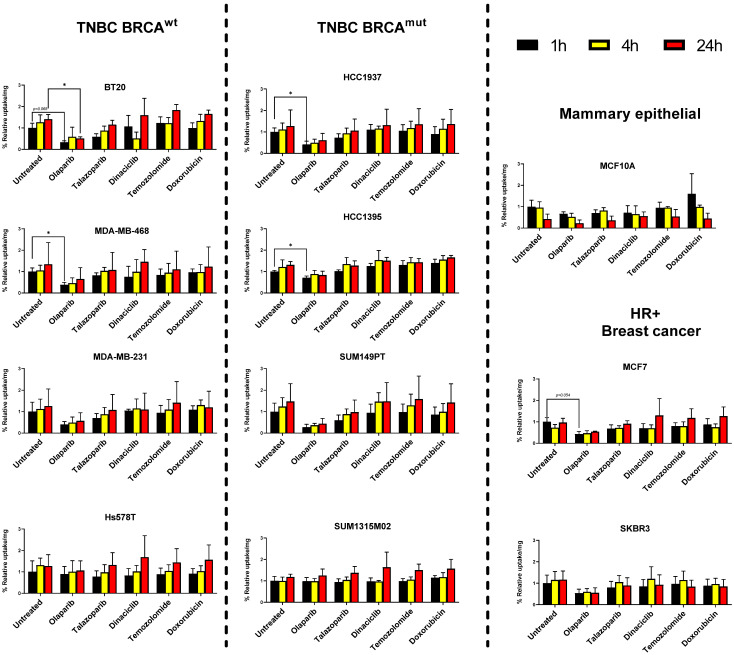
Time dependent uptake of [^125^I]-PARPi-01 (0.5 MBq/mL (for 0.5 × 10^6^ cells i.e., 1 Bq/cell); 1, 4, and 24 h, with black, yellow, and red columns, respectively) upon pre-treatment with different chemotherapeutic strategies, such as olaparib (1 µM, 72 h), talazoparib (10 nM, 72 h), dinaciclib (5 µM, 72 h) temozolomide (50 µM, 48 h) and Dox-NP (doxorubicin, 100 nM, 6 h), in each cell line. % Uptake normalised to the untreated samples at 1 h post incubation. Significance was tested using two-way ANOVA with Dunnet’s post-hoc test (n = 3, * *p* < 0.05).

Several studies report that combination of PARPis with chemotherapeutic drugs show synergistic therapeutic effects [25,26,27]. In this study three chemotherapeutic drugs (Dinaciclib, Temozolomide, Liposomal Doxorubicin) were chosen based on previous studies and evaluated for their potential to affect the [^125^I]-PARPi-01 uptake upon pretreatment in their previously reported dosages. The cells were pre-treated with the cyclin-dependent kinase (CDK) inhibitor dinaciclib (5 µm, 72 h), DNA alkylating temozolomide (50 µM, 48 h) prior to incubation with [^125^I]-PARPi-01. Pre-treatment with the DNA intercalating liposomal doxorubicin (Dox-NP, 100 nM) was done for 6 h prior to treatment with [^125^I]-PARPi-01. Liposomal formulation of doxorubicin was chosen due to the prevalent clinical use of this type of formulation as opposed to Doxorubicin in solution. The cellular uptake and retention were evaluated at 1, 4, and 24 h later. Additionally, [^125^I]-PARPi-01 uptake upon long term exposure to olaparib (1 µM, 72 h) and talazoparib (10 nM, 72 h) were studied. This was performed to assess if a long-term exposure (72 h pre-treatment) has a blocking effect on ^125^I-PARPi-01 uptake or leads to a possible uptake increase due to enhanced PARP1 expression. Due to the enhanced cytotoxicity of talazoparib as compared to olaparib, they were treated at 10 nM and 1 µM, respectively. Remarkably, while a time dependent increase in uptake was observed in almost all cancer cell lines irrespective of pre-treatments (except SKBR3), the mammary epithelial cell line MCF10A showed time dependent decrease despite of detected high cellular PARP1 expression (Figure 2 and Figure S3B). While the short pre-treatment with Dox-NP enhanced uptake in MCF10A, a general time dependent decrease of the cellular tracer accumulation was observed, indicating lack of retention. This can be explained by a possible lack of the enzymatically active PARP1 in MCF10A in contrast to the cancer cell-lines [28].

Long-term exposure (72 h) to the PARPi olaparib (1 µM) was effective in blocking uptake (0.8 − 0.5x) up to 24 h in almost all cell lines, except Hs578T and SUM1315M02. Interestingly, a pre-treatment with the highly affine PARPi talazoparib (10 nM) did not block as effectively as olaparib (0.97 ± 0.26x vs. 0.55 ± 0.16x relative uptake at 24 h, for pre-treatment with talazoparib and olaparib, respectively). Exposure to chemotherapeutic drugs did not show any significant impact on cellular [^125^I]-PARPi-01 uptake. However, the PI analysis of nuclear fractions visualised a high impact of short pre-treatment with Dox-NP on [^125^I]-PARPi-01 nuclear uptake as early as at 1 h and retention up until 24 h specifically in the breast cancer cells indicating short pre-treatment with Dox-NP as potential enhancer in nuclear uptake of [^125^I]-PARPi-01 (Figure S3B). This effect is less significant in the cell uptake results, which is possibly due to the high background activity of membrane bound [^125^I]-PARPi-01 because of its high lipophilicity. However, this effect correlated with an increased PARP1 localisation as detected by immunofluorescence microscopy (Figure S4).

### 3.3. Dox-NP Combination with [^125^I]-PARPi-01 Causes High DNA Damage (γH2AX Foci) in Resistant Breast Cancer Cell Lines

**Figure 3:**
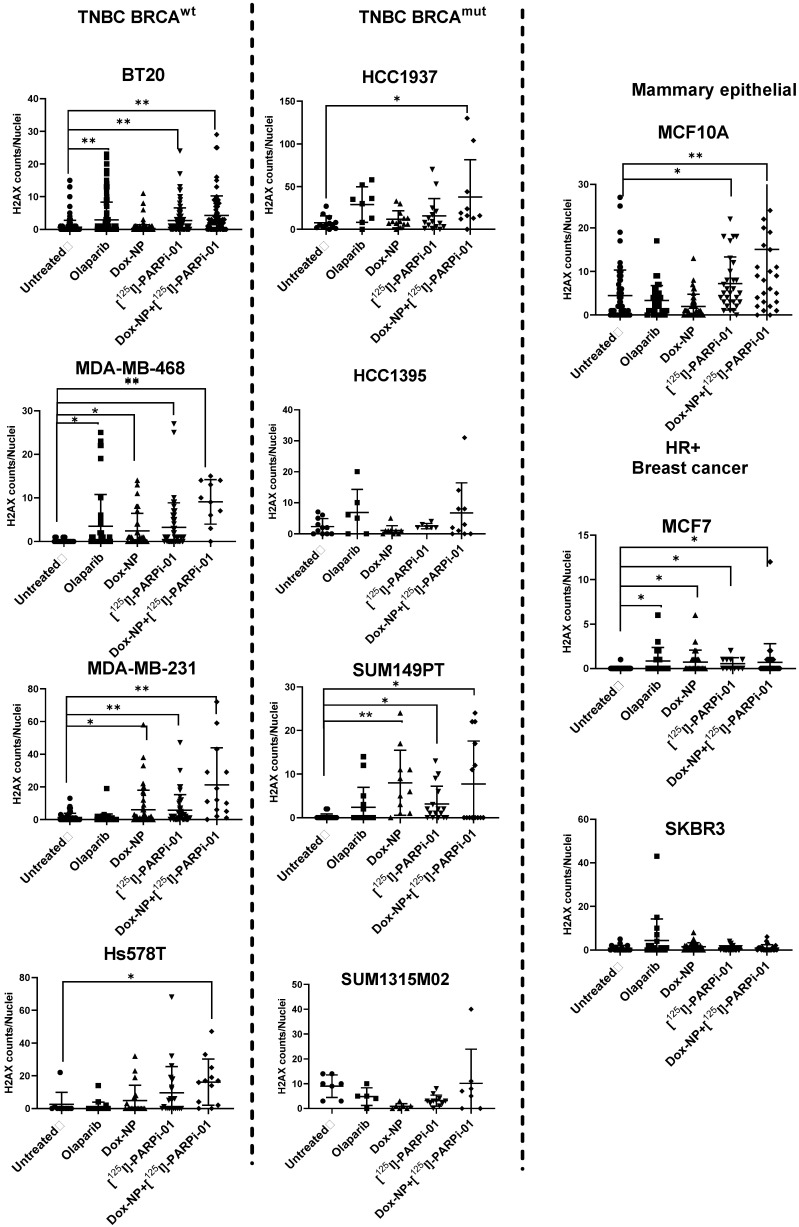
Quantification of γH2AX foci per nucleus in microscopic images upon treatment with olaparib (1 µM, 72 h), Dox-NP (100 nM, 6 h), [^125^I]-PARPi-01 (2.3 MBq/1 mL (for 10^6^ cells, i.e., 2.3 Bq/cell), 72 h), pretreatment with Dox-NP (100 nM, 6 h) followed by [^125^I]-PARPi-01 (2.3 MBq/1 mL (for 10^6^ cells, i.e., 2.3 Bq/cell), 66 h). Significance was analysed using one way ANOVA and Dunn’s post-hoc test (n = 3,* *p* < 0.05; ** *p* < 0.001).

Amongst the BRCA^wt^ cell lines, the epithelial cell-lines (BT20, MDA-MB-468) and the mesenchymal stem cell line (MDA-MB-231) harboured significant dsDNA damage, as observed by the increase in γH2AX foci formation after [^125^I]-PARPi-01 monotherapy. The DNA damage level was further increase upon combination with Dox-NP pre-treatment (Figure 3). However, the treatment refractive cell line Hs578T showed significant dsDNA strand breaks only after the combination treatment. Interestingly, the two epithelial cell lines BT20 and MDA-MB-468 showed DNA damage after olaparib monotherapy proving PARP trapping (PARPi-PARP-DNA complex formation) mediated downstream DNA strand breakages. This confirms PARPi sensitivity of BT20 and MDA-MB-468, which were shown earlier [29,30]. Interestingly, the therapeutic efficacy did not increase by the radiolabelling of olaparib, as additionally visualised by immunofluorescence microscopy (Figure S5). [^125^I]-PARPi-01 monotherapy is seemingly advantageous over treatment with olaparib as observed in the PARPi insensitive MDA-MB-231 and in Hs578T. Enhanced PARP1 expression was observed upon short Dox-NP treatment (100 nM, 6 h). Notably, this effect was not observed uniformly in all cell lines, indicating differences in DNA repair pathways due to their varying underlying genotypes. Amongst the BRCA^mut^ cohort, only the epithelial SUM149PT showed to be responsive with significantly higher dsDNA damage upon [^125^I]-PARPi-01 monotherapy. The epithelial HCC1937 showed significant dsDNA damage induction upon combination treatment. In HCC1395 and SUM1315M02 cell lines, the dsDNA damage level was not significant. The breast cancer cell lines SKBR3 remained unaffected whereas MCF7 did show higher DNA damage upon each treatment. Importantly, even if MCF10A showed significant H2AX formations upon [^125^I]-PARPi-01 monotherapy, the foci amount was lower when compared to that detected in the responsive malignant breast cells.

### 3.4. Pre-Treatment with Dox-NP Significantly Enhances Mitotic Phase Arrest in TNBC Cells

**Figure 4:**
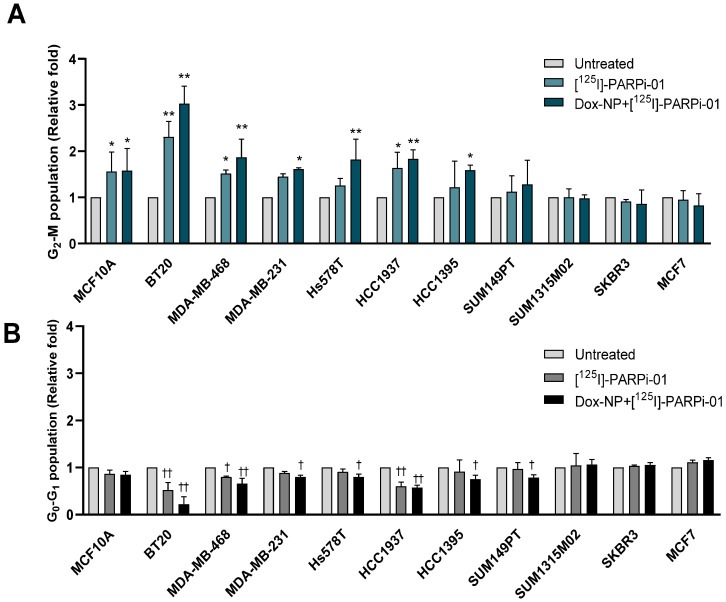
Comparison of mitotic phase (**A**) and growth phase (**B**) population after 72 h of [^125^I]-PARPi-01 (2.24 ± 0.3 MBq/1 mL (10^6^ cells, i.e., 2.24 Bq/Cell)) treatment with or without Dox-NP (100 nM, 6 h) pre-treatment in different cell lines (n = 3). Significance was analysed using two-way ANOVA and Dunnet’s post-hoc test (* *p* < 0.05; ** *p* < 0.001; ^†^ *p* < 0.05; ^††^ *p* < 0.001).

Since the sensitivity to radiation depends on the cell cycle phase, the cell cycle distribution was analysed in each cell line using flow cytometry. After [^125^I]-PARPi-01 mono-treatment (2.4 ± 0.3 MBq/10^6^ cells, 72 h) three Basal A epithelial TNBC cell lines BT20, MDA-MB-468 and HCC1937 showed changes in the cell cycle distribution with a significant increase in the most radiosensitive G2-M phase (*p* < 0.001) (Figure 4A; Table 1). This responsiveness irrespective of BRCA status can be attributed to the homozygous deletion of PTEN (HCC1937 and MDA-MB-468) and PI3CA mutation (BT20), both known to lead to DNA repair deficiency [31]. Three TNBC cell lines belonging to the aggressive Basal B subtype (MDA-MB-231, Hs578T and HCC1395) showed a significant increase in G2-M population upon pre-treatment with Dox-NP (100 nM, 6 h). All responsive cell lines showed a corresponding decrease in the G0–G1 population in their respective treatments (Figure 4B).

SUM149PT (BRCA^mut^) also showed a decrease in the G0–G1 population despite a lack in significant mitotic arrest. The TNBC SUM1315M02 (BRCA^mut^), HER2+/hormone receptor positive (HR+) breast cancer cell lines SKBR3 and MCF7 (BRCA^wt^) did not show significant changes upon treatment with [^125^I]-PARPi-01 all one and in combination with Dox-NP. Additionally, changes in the cell cycle distribution was evaluated for other combination chemotherapy strategies and compared to that induced by an Auger emitter treatment (Figure S6). The two Basal A cell types BT20 and MDA-MB-468 showed significant mitotic phase arrest after mono-therapy with talazoparib (10 nM, 72 h) and in combination with DNA damaging chemotherapies (Dox-NP, TMZ). Additionally, these cell lines showed the therapeutic response to Auger emitter treatment as monotherapy and in combination with Dox-NP (Figure S6A,B). Remarkably high response was seen in the metastatic mesenchymal stem cell like MDA-MB-231 (3.4 times; Figure S6C) and carcinosarcomatous Hs578T (5.4 times; Figure S6D) cell lines to TMZ mono- and in combination with olaparib. This is probably due to their MGMT deficiency and MGMT promoter methylation [32,33]. While MDA-MB-231 showed PARPi response upon talazoparib monotherapy and in combination with chemotherapeutic agents, Hs578T did not show any PARPi sensitivity. Amongst the BRCA^mut^ cell lines the epithelial HCC1937 was responsive to combination treatment of dinaciclib with olaparib and to treatment with Auger emitting PARPi (Figure S6E). HCC1395 showed response to monotherapies with olaparib and talazoparib and to dinaciclib/doxorubicin in combination with olaparib (Figure S6F). The BRCA^mut^ cell lines SUM1315M02 and SUM149PT did not show a significant increase in mitotic arrest in all the studied combination strategies (Figure S6G,H). Amongst non-TNBC breast cancer cells, SKBR3 showed response only to dinaciclib in combination with olaparib. Importantly, for the epithelial breast cells MCF10A no significant changes were detected (Figure S6I). Overall, treatment combining [^125^I]-PARPi-01 with DOX-NP affected the mitotic activity of nearly all investigated TNBC cells. These results indicate that Auger emitting PARP targeting therapy is capable of inducing mitotic arrest in a lower dosage and can alleviate possible systemic toxicity and improve tolerability.

### 3.5. Dox-NP Pretreatment with [^125^I]-PARPi-01 Leads Responsive Cells to Apoptosis

**Figure 5:**
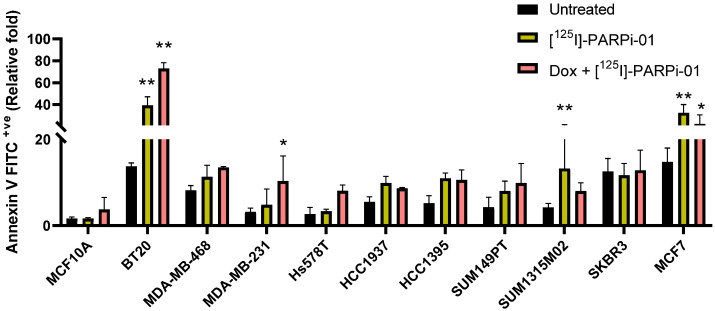
Apoptotic population (Annexin V FITC + ve) in breast cancer cell lines upon treatment with [^125^I]-PARPi-01 (2.24 ± 0.3 MBq/1 mL (10^6^ cells, i.e., 2.24 Bq/Cell)), 72 h) and 6 h Dox pre-treatment 100 nM) with [^125^I]-PARPi-01. Significance was analysed using two-way ANOVA and Dunnet’s post-hoc test (n = 3; * *p* < 0.05, ** *p* < 0.001).

The cytotoxic effect of [^125^I]-PARPi-01 treatment was evaluated by the Annexin V-based apoptosis assay using flow cytometry. Importantly, the benign mammary epithelial cell line did not show a significant increase in apoptosis upon treatment with [^125^I]-PARPi-01 as monotherapy or combined with Dox-NP pre-treatment (Figure 5 and Figure S7I). The most responsive BRCA^wt^ cell line BT20 showed significant increase in apoptosis upon [^125^I]-PARPi-01 mono-treatment (Figure 5 and Figure S7A). Moreover, a pre-treatment with Dox-NP (100 nM, 6 h) further increased the apoptotic response. This synergistic effect was also detected in MDA-MB-231 and Hs578T cells (Figure 5 and Figure S7C,D). Among the BRCA^mut^ cell lines, only SUM1315M02 exhibited an increase in Annexin V positive population after monotherapy with [^125^I]-PARPi-01 despite no effect seen in cell cycle analysis. However, there was no synergistic effect of pre-treatment with DOX-NP. Moreover, this was not reflected in late apoptosis (Figure S7I). The combination of the pan-CDK inhibitor dinaciclib (5 nM) with olaparib (1 µM) caused significant apoptotic induction in the Myc overexpressing SUM1315M02 indicating dinaciclib as a potent combination strategy for Myc overexpressing cell types. Although MDA-MB-468 did not show significant increase in overall apoptosis, comparison of different treatments showed significant increase in early apoptosis upon combination of Dox-NP with [^125^I]-PARPi-01 (Figure S7B). Interestingly, the increase in apoptosis in other cell lines (HCC1937, HCC1395) showing a response in mitotic arrest was not significantly high. This might be due to the protection of DNA replication fork mediated resistance to PARP1 inhibitors [34]. However, it is evident that BRCA status of cells is irrelevant to responsiveness to PARPi single and combination treatment. Concurrent to the observation in mitotic phase arrest, the BRC^wt^ cell line Hs578T showed significant apoptotic induction after combined treatment with TMZ + ola due to its lack of MGMT expression (Figure S7D). Table 1 summarises the quantitative effects observed in cell cycle and apoptosis assays after treatment with [^125^I]-PARPi-01 as single treatment or combined with Dox-NP pre-treatment.

### 3.6. [^125^I]-PARPi-01 Significantly Reduces Tumorigenicity in All Colony Forming Cancer Cell Lines

**Figure 6:**
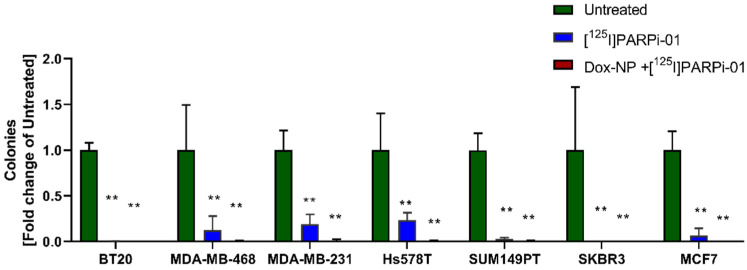
Soft agar colony formation. Reduction in colony formation upon treatment with [^125^I]-PARPi-01 (2.345 ± 0.43 MBq/1 mL (in 10^6^ cells i.e., 2.345 Bq/cell), 72 h) as single and in combination with Dox-NP (6 h, 100 µM) pre-treatment is plotted as fold change in comparison to untreated cell lines. Statistical analysis was done using two-way ANOVA and Dunnet’s post-hoc test (n = 3; ** = *p* < 0.0001).

The potential for anchorage independent growth of all cancer cell lines were evaluated using soft agar assay. The untreated cell line BT20 exhibited most aggressive colony formation (at 21 days) with colony diameters >250 µm (Figure S8). This high tumourigenicity was significantly reduced upon treatment with [^125^I]-PARPi-01 (2.345 ± 0.43 MBq/10^6^ cells) as monotherapy and further completely inhibited after combined pre-treatment with Dox-NP (Figure 6). Similarly, MDA-MB-468 also showed colonies with diameters of >125 µm, which were significantly reduced upon mono-therapy and totally inhibited upon treatment with combination therapy. The resistant mesenchymal MDA-MB-231 and sarcomatous Hs578T cell lines significantly reduced soft agar colony formation for both, mono-therapy and combination therapy approaches. Unlike MDA-MB-468, MDA-MB-231 and Hs578T showed formation of colonies with diameter >125 µm upon monotherapy although significantly lower. This can be explained by the presence of mutant KRas (KRAS^mut^) and mutant HRas (HRas^mut^) genes in MDA-MB-231 and Hs578T, respectively, which are known to drive the epithelial–mesenchymal invasion mediated tumourigenicity (Figure S8) [35,36,37]. Amongst the BRCA^mut^ cell lines, SUM149PT showed colony formation with colony diameter ranging between 50 and 80 µm, with complete inhibition of malignant phenotype upon mono/combination therapy. Other non-TNBC cell lines MCF7 and SKBR3 also exhibited significantly lower colony numbers and loss of the tumorigenic potential upon mono/combination therapies. The mammary epithelial cell line MCF10A and other BRCA^mut^ cell lines HCC1937, HCC1395, and SUM1315M02 did not show colony formation (Figure S8), possibly due to requirements of higher seeding densities or higher soft-agar percentage formulation [38,39]. Taken together, this experiment proves that [^125^I]-PARPi-01 mediated Auger emitter therapy effectively reduces the tumourigenicity of breast cancer cells in vitro.

## 4. Discussion

PARP1 inhibitors (PARPis) are clinically approved for metastatic breast cancers and are targeted to the nuclear protein PARP. The maximal tolerated dose (MTD) of the PARPi olaparib (400 mg BID) has been reported to induce side effects. Moreover, for patients receiving extensive platinum-based pre-treatment and PARPi, a development of myelodysplastic syndrome (MDS) and acute myeloid leukaemia (AML) has also been reported [40]. Besides side effects, although PARPis are approved for BRCA^mut^ TNBC patients, several BRCA^mut^ patients are still unresponsive warranting PARPi sensitivity based stratification [41].

Auger emitter (AE) based therapy strategies have gained recognition and are studied in several cancers [42,43,44,45]. Due to their advantageous property of high linear energy transfer at a short range, AEs are of therapeutic value when delivered in close proximity to DNA. Due to the nuclear location of PARP, PARPis are ideal for radiolabelling with Auger emitters to deliver them close to DNA, thereby lending promising therapeutic value. Therefore, at the molecular level the AE radiolabelled PARPis bind and block the PARP1 mediated DNA repair, along with causing further DNA damage contributing to a two-hit therapy approach. Currently, Auger emitter radionuclide ^123/125^I have been radiolabelled in two FDA-approved PARPi backbones - olaparib: [^123^I]-MAPi (structurally similar to our probe) [24] and Rucaparib: [^123/125^I]-KX1 [46]. During the course of this study, preclinical therapeutic evaluation of ^123^I-MAPi was reported in glioblastoma models, in which a significant survival advantage was shown upon delivering the [^123^I]-MAPi via local intratumoral injection or delivery. Similarly, ^125^I-KX1 was tested in vitro in ovarian cancer cell lines and showed reduction in cell survival.

In this study, we reported the synthesis of a PARP1 specific, olaparib derived Auger emitting PARPi: [^125^I]-PARPi-01. Our radiosynthesis yielded up to 2.5 times higher molar activity and significantly higher radiochemical yield than reported by Wilson et al. [24]. Here, we demonstrate an efficient induction of highly damaging DNA double strand break (γH2AX foci) and significantly reduced tumorigenicity, upon treatment (72 h) with [^125^I]PARPi-01, at molar concentrations of 0.01–0.04 µM. Importantly, it is 250–1000 times lower than minimal cytotoxic concentration of olaparib proving amplified therapeutic efficiency in the clinic [30]. This will allow chemical dosage reduction without loss or impairment of the therapeutic efficiency.

This AE-based PARPi: [^125^I]-PARPi-01 can be of therapeutic value in TNBC. Nevertheless, TNBC is highly heterogenous and can show varied response. Due to this, a tailored approach based on clear understanding of subtype-specific response is crucial [47]. The observed significant reduction in tumorigenicity in all tested cancer cell lines proves the potential of combined PARPi Auger emitter strategy irrespective of subtype. However, mitotic arrest results show that Basal A epithelial sub-types respond to [^125^I]-PARPi-01 monotherapy. The most radiosensitive cell-cycle phase is the G2-M phase and enhanced cell cycle arrest in this phase shows that the Auger emitter functions by being recruited to the PARP1 mediated DNA repair machinery possibly in the S-phase followed by further DNA damage and arrest in the G2-M phase.

Clearly, as has been reported in several studies, BRCA mutation status alone does not play a role in the response to PARP inhibition [48]. Their genetic background with an impaired homologous recombination (homologous recombination deficiency or HRD) or DNA repair efficiency is probably the reason for the responsiveness of these cell lines.

Among the cell lines investigated in this study, BRCA^wt^ epithelial cells BT20 and MDA-MB-468 have shown the most promising therapeutic response as visualised by [^125^I]-PARPi-01 induced dsDNA damage, significant cell cycle arrest and reduction in clonogenic survival. As shown in other studies, these cell lines are PARPi sensitive, which resulted in olaparib mediated enhanced γH2AX formation and mitotic phase arrest upon PARPi mono (talazoparib) and combination therapies as presented here [30]. This explains the detected [^125^I]-PARPi-01 sensitivity in BT20 [29]. Similarly, MDA-MB-468 have a heavy deficiency of NER pathway that possibly contributes additionally to the PARPi sensitivity [13,49]. However, the therapeutic effect can be attributed mainly to their underlying lack of DNA repair to rescue Auger emitter induced DNA damage. The high telomeric allelic imbalance (TAI) impairs the DNA repair [50]. TAI also contributes to homologous recombination deficiency making the cells sensitive not only to PARPis, but also to genotoxic chemotherapy and therefore ideal responders for our two-hit therapy approach. The absence of the tumour suppressor gene PTEN is known to contribute to HR repair and genomic instability, and its deletion/loss-of-function has proven to render cells sensitive to PARPi mediated synthetic lethality [51,52]. In spite of the presence of an advantageous PTEN homozygous deletion, HCC1937 has been reported to show less to no response to PARPi-based synthetic lethality, but sensitive to cisplatin mediated DNA damage [30,39]. In our study, we observed similar insensitivity to PARPi, however a significant response to Auger emitter-based therapy in mitotic arrest and block of colony formation. This confirms the advantage of [^125^I]-PARPi-01 based PARP inhibition coupled with DNA damaging approach. Thus, HRD positive patients harbouring somatic/germline PTEN deletion/loss-of-function can clearly benefit from targeted [^125^I]-PARPi-01 therapy.

Among the Basal B cell lines, metastatic mesenchymal like TNBC cell lines (MDA-MB-231 (BRCA^wt^), Hs578T (BRCA^wt^) and HCC1395 (BRCA^mut^)), MDA-MB-231, and HCC1395 showed talazoparib sensitivity, accordingly to previous studies [30,53]. Both MDA-MB-231 and Hs578T showed enhanced apoptosis and reduction of colony formation upon combination therapy strategy. This correlates with the Dox-NP induced enhanced uptake and retention of [^125^I]-PARPi-01 observed in uptake assay. However, their MGMT deficiency and response to TMZ predisposes these subtypes for TMZ combination with [^125^I]-PARPi-01 treatment [33,54]. Among the above mentioned cell lines the BRCAness signature has been reported to be low for BT20 and MDA-MB-231, which have still shown promising response to Auger emitter-based therapy [55]. Other Basal B non-responsive TNBC cell lines include SUM149PT and SUM1315M02. Although SUM149PT has shown reduction in colony formation, it has shown to possess functional homologous recombination machinery, contributing to its lack of therapeutic response in spite of the significant H2AX formation [56]. Similarly SUM1315M02 has shown to resist DNA damaging drugs and possess homologous recombination [56,57].

Therefore, apart from BRCA status, overall BRCAness, homologous recombination proficiency, and underlying DNA repair deficiency due to TAI are all helpful for stratifying PARPi mediated Auger emitter treatment response. Further dosimetry studies will provide data for estimation of the minimum dosage required for better therapeutic responsiveness for each TNBC subtype regardless the genomic phenotype. Recent clinical studies using olaparib monotherapy for TNBC showed promising response in HRD high patients [58]. Among the current HRD screening tests in clinic only two (Myriad’s myChoice^®^ CDx and HRD Focus Panel) screen for TAI in patient samples to account for HRD [59].

Taken together these results show that [^125^I]-PARPi-01 mono-therapy significantly induces γH2AX formation, mitotic phase arrest higher than olaparib and blocks the tumorigenicity in TNBC cells regardless of BRCA status, PI3K mutations and RAS family mutations. In resistant subtypes, therapy combining [^125^I]-PARPi-01 with doxorubicin pre-treatment showed enhanced killing efficacy with a complete inhibition of colony formation. The general therapeutic efficacy is remarkable, considering the apoptosis rates detected in some cell lines. This underlies the unique therapeutic potential of Auger electron emitters and of the presented approach combining the endogenous irradiation and PARP inhibition. Therefore, Auger emitter-based [^125^I]-PARPi-01 therapy provides a promising and attractive alternative to conventional chemotherapy for patients stratified to have better prognosis with PARP1 monotherapy or in combination with DNA damaging agents. Owing to its high efficacy and low required dosage, [^125^I]-PARPi-01 is expected to induce less systemic toxicity.

## 5. Conclusions

In this study, we developed and evaluated an Auger emitter-based therapeutic PARP inhibitor [^125^I]-PARPi-01, derived from the clinical PARP inhibitor olaparib, and assessed its therapeutic value in different TNBC subtypes. Apart from the ability of [^125^I]-PARPi-01 to cause DNA damage at (up to) significantly lower concentrations than olaparib, we identified responsive subtypes of TNBC. Responsive subtypes with underlying impaired DNA repair mechanism are forced towards apoptosis upon treatment with [^125^I]-PARPi-01, while the benign mammary epithelial cell line is not affected significantly. For the non-responsive TNBC subtypes, a combination therapy strategy involving Dox-NP as conditioning for treatment with [^125^I]-PARPi-01 significantly increased the therapeutic response, resulting in a complete inhibition of the clonogenic growth. Thus, an Auger-emitting inhibitor of PARP is a promising therapeutic strategy that can be (optimally and individually) fitted by combined pre-treatment, accordingly, to the unique phenotype of each single TNBC subtype.

## Figures and Tables

**Table 1:** Fold change of mitotic arrest and annexin V induction upon treatment with [^125^I]-PARPi-01 or Dox+[125]I-PARPi-01. Cell lines showing therapeutic response in either mitotic arrest or apoptosis induction are highlighted in red.

		[^125^I]-PARPi-01		Dox+[^125^I]-PARPi-01	
		G2-M Arrest	Annexin V + ve	G2-M Arrest	Annexin V + ve
Control	MCF10A	1.5 ± 0.4	1 ± 0.12	1.6 ± 0.5	2.27 ± 1.68
TNBC BRCA^wt^	BT20	2.3 ± 0.3	2.86 ± 0.57	3.1 ± 0.4	5.30 ± 0.40
	MDA-MB-468	1.5 ± 0.1	1.38 ± 0.33	1.9 ± 0.4	1.65 ± 0.02
	MDA-MB-231	1.4 ± 0.1	1.53 ± 1.14	1.6 ± 0.1	3.24 ± 1.84
	Hs578T	1.2 ± 0.1	1.25 ± 0.16	1.8 ± 0.4	3 ± 0.5
TNBC BRCA^mut^	HCC1937	1.6 ± 0.3	1.79 ± 0.27	1.8 ± 0.2	1.56 ± 0.03
	HCC1395	1.1 ± 0.7	2.09 ± 0.31	1.6 ± 0.1	1.88 ± 0.39
	SUM149PT	1.1 ± 0.3	1.88 ± 0.54	1.3 ± 0.5	2.31 ± 1.06
	SUM1315M02	1.0 ± 0.2	3.16 ± 2.02	1.0 ± 0.1	1.91 ± 0.46
HER2^+ve^ BRCA^wt^	SKBR3	0.9 ± 0.1	0.93 ± 0.22	0.9 ± 0.3	1.02 ± 0.37
	MCF7	0.8 ± 0.1	2.15 ± 0.70	0.8 ± 0.3	1.23 ± 0.36

## Data Availability

The data presented in this study are available on request from the corresponding author.

## Supplementary Materials

The following are available online at https://www.mdpi.com/article/10.3390/cancers14010230/s1. Figure S1A: ^1^H NMR spectrum of 4-{3′-[4″-(4‴-iodobenzoyl)piperazine-1″-carbonyl]-4′-fluorobenzyl}-2H-phthalazin-1-one; Figure S1B: ^13^C{^1^H} NMR spectrum of 4-{3′-[4″-(4‴-iodobenzoyl)piperazine-1″-carbonyl]-4′-fluorobenzyl}-2H-phthalazin-1-one; Figure S1C: ^1^H NMR spectrum of 4-{3′-[4″-(4‴-tributylstannylbenzoyl)piperazine-1″-carbonyl]-4′-fluorobenzyl}-2H-phthalazin-1-one; Figure S1D: ^13^C{^1^H} NMR spectrum of 4-{3′-[4″-(4‴-tributylstannylbenzoyl)piperazine-1″-carbonyl]-4′-fluorobenzyl}-2H-phthalazin-1-one; Figure S2A: Quality control of [125I]-PARPi-01 using gradient Reverse Phase HPLC; Figure S2B: Comparison of 125I vs [125I]-PARPi-01 in TLC; Figure S3A: Western blot analysis of PARP1 expression; Figure S3B: Phosphor-imager analysis of radioactivity in nuclear lysates; Figure S4: Immunofluorescence microscopy of PARP1 expression; Figure S5: Representative Immunofluorescence microscopy of BT20 cells; Figure S6: Comparison of cell cycle phases; Figure S7: Comparison of early and late apoptotic populations and live cell population; Figure S8: Images of colony formation of cell-lines.

## References

[1] Siegel R.L., Miller K.D., Fuchs H.E., Jemal A. (2021). Cancer Statistics. CA Cancer J. Clin..

[2] Schmid P., Adams S., Rugo H.S., Schneeweiss A., Barrios C.H., Iwata H., Diéras V., Hegg R., Im S.-A., Shaw Wright G. (2018). Atezolizumab and Nab-Paclitaxel in Advanced Triple-Negative Breast Cancer. N. Engl. J. Med..

[3] Cortes J., Cescon D.W., Rugo H.S., Nowecki Z., Im S.A., Yusof M.M., Gallardo C., Lipatov O., Barrios C.H., Holgado E. (2020). Pembrolizumab plus chemotherapy versus placebo plus chemotherapy for previously untreated locally recurrent inoperable or metastatic triple-negative breast cancer (KEYNOTE-355): A randomised, placebo-controlled, double-blind, phase 3 clinical trial. Lancet.

[4] Bardia A., Mayer I.A., Vahdat L.T., Tolaney S.M., Isakoff S.J., Diamond J.R., O’Shaughnessy J., Moroose R.L., Santin A.D., Abramson V.G. (2019). Sacituzumab Govitecan-hziy in Refractory Metastatic Triple-Negative Breast Cancer. N. Engl. J. Med..

[5] Seligson J.M., Patron A.M., Berger M.J., Harvey R.D., Seligson N.D. (2020). Sacituzumab Govitecan-hziy: An Antibody-Drug Conjugate for the Treatment of Refractory, Metastatic, Triple-Negative Breast Cancer. Ann. Pharmacother..

[6] Robson M., Im S.-A., Senkus E., Xu B., Domchek S.M., Masuda N., Delaloge S., Li W., Tung N., Armstrong A. (2017). Olaparib for Metastatic Breast Cancer in Patients with a Germline *BRCA* Mutation. N. Engl. J. Med..

[7] Litton J.K., Rugo H.S., Ettl J., Hurvitz S.A., Gonçalves A., Lee K.-H., Fehrenbacher L., Yerushalmi R., Mina L.A., Martin M. (2018). Talazoparib in Patients with Advanced Breast Cancer and a Germline BRCA Mutation. N. Engl. J. Med..

[8] Lyons T.G. (2019). Targeted Therapies for Triple-Negative Breast Cancer. Curr. Treat. Options Oncol..

[9] Gupta G.K., Collier A.L., Lee D., Hoefer R.A., Zheleva V., van Reesema L.L.S., Tang-Tan A.M., Guye M.L., Chang D.Z., Winston J.S. (2020). Perspectives on triple-negative breast cancer: Current treatment strategies, unmet needs, and potential targets for future therapies. Cancers.

[10] Liedtke C., Mazouni C., Hess K.R., André F., Tordai A., Mejia J.A., Symmans W.F., Gonzalez-Angulo A.M., Hennessy B., Green M. (2008). Response to neoadjuvant therapy and long-term survival in patients with triple-negative breast cancer. J. Clin. Oncol..

[11] Pistelli M., Pagliacci A., Battelli N., Santinelli A., Biscotti T., Ballatore Z., Berardi R., Cascinu S. (2013). Prognostic factors in early-stage triple-negative breast cancer: Lessons and limits from clinical practice. Anticancer Res..

[12] Kalimutho M., Parsons K., Mittal D., López J.A., Srihari S., Khanna K.K. (2015). Targeted Therapies for Triple-Negative Breast Cancer: Combating a Stubborn Disease. Trends Pharmacol. Sci..

[13] Chaudhuri A.R., Nussenzweig A. (2017). The multifaceted roles of PARP1 in DNA repair and chromatin remodelling. Nat. Rev. Mol. Cell Biol..

[14] Chalakur-Ramireddy N.K.R., Pakala S.B. (2018). Combined drug therapeutic strategies for the effective treatment of Triple Negative Breast Cancer. Biosci. Rep..

[15] Pogoda K., Niwińska A., Sarnowska E., Nowakowska D., Jagiełło-Gruszfeld A., Siedlecki J., Nowecki Z. (2020). Effects of BRCA Germline Mutations on Triple-Negative Breast Cancer Prognosis. J. Oncol..

[16] Anders C.K., Winer E.P., Ford J.M., Dent R., Silver D.P., Sledge G.W., Carey L.A. (2010). Poly(ADP-ribose) polymerase inhibition: “Targeted” therapy for triple-negative breast cancer. Clin. Cancer Res..

[17] Beniey M., Haque T., Hassan S. (2019). Translating the role of PARP inhibitors in triple-negative breast cancer. Oncoscience.

[18] Gelmon K.A., Tischkowitz M., Mackay H., Swenerton K., Robidoux A., Tonkin K., Hirte H., Huntsman D., Clemons M., Gilks B. (2011). Olaparib in patients with recurrent high-grade serous or poorly differentiated ovarian carcinoma or triple-negative breast cancer: A phase 2, multicentre, open-label, non-randomised study. Lancet Oncol..

[19] Adelstein S.J., Kassis A.I. (1996). Strand breaks in plasmid DNA following positional changes of Auger-electron-emitting radionuclides. Acta Oncol..

[20] Unverricht-Yeboah M., Holtmann K., Kriehuber R. (2020). Comet Assay analysis of DNA strand breaks after exposure to the DNA-incorporated Auger Electron Emitter Iodine-125. Int. J. Radiat. Biol..

[21] Balagurumoorthy P., Xu X., Wang K., Adelstein S.J., Kassis A.I., Balagurumoorthy P. (2012). Effect of distance between decaying 125 I and DNA on Auger-electron induced double-strand break yield. Int. J. Radiat. Biol..

[22] Ku A., Facca V.J., Cai Z., Reilly R.M. (2019). Auger electrons for cancer therapy—A review. EJNMMI Radiopharm. Chem..

[23] Terraneo N., Jacob F., Dubrovska A., Grünberg J. (2020). Novel Therapeutic Strategies for Ovarian Cancer Stem Cells. Front. Oncol..

[24] Wilson T.C., Jannetti S.A., Guru N., Pillarsetty N., Reiner T., Pirovano G. (2020). Improved radiosynthesis of 123I-MAPi, an Auger theranostic agent. Int. J. Radiat. Biol..

[25] Murai J., Zhang Y., Morris J., Ji J., Takeda S., Doroshow J.H., Pommier Y. (2014). Rationale for poly(ADP-ribose) polymerase (PARP) inhibitors in combination therapy with camptothecins or temozolomide based on PARP trapping versus catalytic inhibition. J. Pharmacol. Exp. Ther..

[26] Del Conte G., Sessa C., von Moos R., Viganò L., Digena T., Locatelli A., Gallerani E., Fasolo A., Tessari A., Cathomas R. (2014). Phase I study of Olaparib in combination with liposomal doxorubicin in patients with advanced solid tumours. Br. J. Cancer.

[27] Carey J.P.W., Karakas C., Bui T., Chen X., Vijayaraghavan S., Zhao Y., Wang J., Mikule K., Litton J.K., Hunt K.K. (2017). Synthetic Lethality of PARP Inhibitors in Combination with MYC Blockade Is Independent of BRCA Status in Triple-Negative Breast Cancer. Cancer Res..

[28] Xiao G., Lundine D., Annor G.K., Canar J., Ellison V., Polotskaia A., Donabedian P.L., Reiner T., Khramtsova G.F., Olopade O.I. (2020). Gain-of-function mutant p53 R273H interacts with replicating DNA and PARP1 in breast cancer. Cancer Res..

[29] Węsierska-Gądek J., Mauritz M., Mitulovic G., Cupo M. (2015). Differential Potential of Pharmacological PARP Inhibitors for Inhibiting Cell Proliferation and Inducing Apoptosis in Human Breast Cancer Cells. J. Cell. Biochem..

[30] Keung M.Y., Wu Y., Badar F., Vadgama J.V. (2020). Response of Breast Cancer Cells to PARP Inhibitors Is Independent of BRCA Status. J. Clin. Med..

[31] Minami A., Nakanishi A., Ogura Y., Kitagishi Y., Matsuda S. (2014). Connection between tumor suppressor BRCA1 and PTEN in damaged DNA repair. Front. Oncol..

[32] Wojdacz T.K., Dobrovic A. (2007). Methylation-sensitive high resolution melting (MS-HRM): A new approach for sensitive and high-throughput assessment of methylation. Nucleic Acids Res..

[33] Paranjpe A., Bailey N.I., Konduri S., Bobustuc G.C., Ali-Osman F., Yusuf M.A., Punganuru S.R., Madala H.R., Basak D., Mostofa A.G.M. (2016). New insights into estrogenic regulation of O6-methylguanine DNA-methyltransferase (MGMT) in human breast cancer cells: Co-degradation of ER-α and MGMT proteins by fulvestrant or O6-benzylguanine indicates fresh avenues for therapy. J. Biomed. Res..

[34] Clements K.E., Thakar T., Nicolae C.M., Liang X., Wang H.G., Moldovan G.L. (2018). Loss of E2F7 confers resistance to poly-ADP-ribose polymerase (PARP) inhibitors in BRCA2-deficient cells. Nucleic Acids Res..

[35] Kodaz H., Hastanesi A.E., Klinigi T.O., Kostek O., Bekir Hacioglu M., Erdogan B., Elpen Kodaz C., Hacibekiroglu I., Turkmen E., Uzunoglu S. (2017). Frequency of Ras Mutations (Kras, Nras, Hras) in Human Solid Cancer. EJMO.

[36] Patra S., Young V., Llewellyn L., Senapati J.N., Mathew J. (2017). BRAF, KRAS and PIK3CA mutation and sensitivity to Trastuzumab in breast cancer cell line model. Asian Pac. J. Cancer Prev..

[37] Koh M.S., Moon A. (2011). Activation of H-Ras and Rac1 correlates with epidermal growth factor-induced invasion in Hs578T and MDA-MB-231 breast carcinoma cells. Biochem. Biophys. Res. Commun..

[38] Wu X., Zahari M.S., Renuse S., Kelkar D.S., Bharbuiya M.A., Rojas P.L., Stearns V., Gabrielson E., Malla P., Sukumar S. (2017). The non-receptor tyrosine kinase TNK2/ACK1 is a novel therapeutic target in triple negative breast cancer. Oncotarget.

[39] Tassone P., Tagliaferri P., Perricelli A., Blotta S., Quaresima B., Martelli M.L., Goel A., Barbieri V., Costanzo F., Boland C.R. (2003). BRCA1 expression modulates chemosensitivity of BRCA1-defective HCC1937 human breast cancer cells. Br. J. Cancer.

[40] Sachdev E., Tabatabai R., Roy V., Rimel B.J., Mita M.M. (2019). PARP Inhibition in Cancer: An Update on Clinical Development. Target. Oncol..

[41] McGrail D.J., Lin C.C.-J., Garnett J., Liu Q., Mo W., Dai H., Lu Y., Yu Q., Ju Z., Yin J. (2017). Improved prediction of PARP inhibitor response and identification of synergizing agents through use of a novel gene expression signature generation algorithm. NPJ Syst. Biol. Appl..

[42] Shen C.J., Minn I., Hobbs R.F., Chen Y., Josefsson A., Brummet M., Banerjee S.R., Brayton C.F., Mease R.C., Pomper M.G. (2020). Auger radiopharmaceutical therapy targeting prostate-specific membrane antigen in a micrometastatic model of prostate cancer. Theranostics.

[43] Rosenkranz A.A., Slastnikova T.A., Karmakova T.A., Vorontsova M.S., Morozova N.B., Petriev V.M., Abrosimov A.S., Khramtsov Y.V., Lupanova T.N., Ulasov A.V. (2018). Antitumor activity of Auger electron emitter 111In delivered by modular nanotransporter for treatment of bladder cancer with EGFR overexpression. Front. Pharmacol..

[44] Osytek K.M., Blower P.J., Costa I.M., Smith G.E., Abbate V., Terry S.Y.A. (2021). In vitro proof of concept studies of radiotoxicity from Auger electron-emitter thallium-201. EJNMMI Res..

[45] Pirovano G., Jannetti S.A., Carter L.M., Sadique A., Kossatz S., Guru N., Demétrio De Souza França P., Maeda M., Zeglis B.M., Lewis J.S. (2020). Targeted brain tumor radiotherapy using an Auger emitter. Clin. Cancer Res..

[46] Riad A., Gitto S.B., Lee H., Winters H.D., Martorano P.M., Hsieh C.-J., Xu K., Omran D.K., Powell D.J., Mach R.H. (2020). PARP Theranostic Auger Emitters Are Cytotoxic in BRCA Mutant Ovarian Cancer and Viable Tumors from Ovarian Cancer Patients Enable Ex-Vivo Screening of Tumor Response. Molecules.

[47] Manjunath M., Choudhary B. (2021). Triple-negative breast cancer: A run-through of features, classification and current therapies. Oncol. Lett..

[48] Varanda A.B., Martins-Logrado A., Ferreira M.G., Fior R. (2020). Zebrafish xenografts unveil sensitivity to Olaparib beyond BRCA status. Cancers.

[49] Rajkumar-Calkins A.S., Szalat R., Dreze M., Khan I., Frazier Z., Reznichenkov E., Schnorenberg M.R., Tsai Y.-F., Nguyen H., Kochupurakkal B. (2019). Functional Profiling of Nucleotide Excision Repair in Breast Cancer. DNA Repair.

[50] Birkbak N.J., Wang Z.C., Kim J.-Y., Eklund A.C., Li Q., Tian R., Bowman-Colin C., Li Y., Greene-Colozzi A., Iglehart J.D. (2012). Telomeric allelic imbalance indicates defective DNA repair and sensitivity to DNA-damaging agents. Cancer Discov..

[51] Mansour W.Y., Tennstedt P., Volquardsen J., Oing C., Kluth M., Hube-Magg C., Borgmann K., Simon R., Petersen C., Dikomey E. (2018). Loss of PTEN-assisted G2/M checkpoint impedes homologous recombination repair and enhances radio-curability and PARP inhibitor treatment response in prostate cancer. Sci. Rep..

[52] Mendes-Pereira A.M., Martin S.A., Brough R., McCarthy A., Taylor J.R., Kim J.S., Waldman T., Lord C.J., Ashworth A. (2009). Synthetic lethal targeting of PTEN mutant cells with PARP inhibitors. EMBO Mol. Med..

[53] Holme H., Gulati A., Brough R., Fleuren E.D.G., Bajrami I., Campbell J., Chong I.Y., Costa-Cabral S., Elliott R., Fenton T. (2018). Chemosensitivity profiling of osteosarcoma tumour cell lines identifies a model of BRCAness. Sci. Rep..

[54] Shen B., Chapman J.H., Custance M.F., Tricola G.M., Jones C.E., Furano A.V. (2020). Perturbation of base excision repair sensitizes breast cancer cells to APOBEC3 deaminase-mediated mutations. Elife.

[55] Teraoka S., Muguruma M., Takano N., Miyahara K., Kawate T., Kaise H., Yamada K., Miyazawa K., Ishikawa T. (2020). Association of BRCA Mutations and BRCAness Status With Anticancer Drug Sensitivities in Triple-Negative Breast Cancer Cell Lines. J. Surg. Res..

[56] Gu Y., Helenius M., Väänänen K., Bulanova D., Saarela J., Sokolenko A., Martens J., Imyanitov E., Kuznetsov S. (2016). BRCA1-deficient breast cancer cell lines are resistant to MEK inhibitors and show distinct sensitivities to 6-thioguanine OPEN. Nat. Publ. Gr..

[57] Gu Y., Wang C., Zhu R., Yang J., Yuan W., Zhu Y., Zhou Y., Qin N., Shen H., Ma H. (2021). The cancer-testis gene, MEIOB, sensitizes triple-negative breast cancer to PARP1 inhibitors by inducing homologous recombination deficiency. Cancer Biol. Med..

[58] Eikesdal H.P., Yndestad S., Elzawahry A., Llop-Guevara A., Gilje B., Blix E.S., Espelid H., Lundgren S., Geisler J., Vagstad G. (2021). Olaparib monotherapy as primary treatment in unselected triple negative breast cancer. Ann. Oncol..

[59] Wagener-Ryczek S., Merkelbach-Bruse S., Siemanowski J. (2021). Biomarkers for homologous recombination deficiency in cancer. J. Pers. Med..

