# Correction: Sankaranarayanan et al. Auger Emitter Conjugated PARP Inhibitor for Therapy in Triple Negative Breast Cancers: A Comparative In-Vitro Study. *Cancers* 2022, *14*, 230

**DOI:** 10.3390/cancers15092641

**Published:** 2023-05-06

**Authors:** Ramya Ambur Sankaranarayanan, Jennifer Peil, Andreas T. J. Vogg, Carsten Bolm, Steven Terhorst, Arno Classen, Matthias Bauwens, Jochen Maurer, Felix Mottaghy, Agnieszka Morgenroth

**Affiliations:** 1 Department of Nuclear Medicine, University Hospital Aachen, RWTH Aachen University, 52074 Aachen, Germany; rambursankar@ukaachen.de (R.A.S.); jennifer.peil@uk-koeln.de (J.P.); avogg@ukaachen.de (A.T.J.V.); matthias.bauwens@mumc.nl (M.B.); fmottaghy@ukaachen.de (F.M.); 2 Institute of Organic Chemistry, RWTH Aachen University, 52056 Aachen, Germany; carsten.bolm@oc.rwth-aachen.de (C.B.); steven.terhorst@rwth-aachen.de (S.T.); arno.classen@oc.rwth-aachen.de (A.C.); 3 Department of Radiology and Nuclear Medicine, Maastricht University Medical Center (MUMC+), 6229HX Maastricht, The Netherlands; 4 School of Nutrition and Translational Research in Metabolism (NUTRIM), Maastricht University, 6229HX Maastricht, The Netherlands; 5 Department of Gynecology and Obstetrics, University Hospital Aachen, RWTH Aachen University, 52074 Aachen, Germany; jmaurer@ukaachen.de

The authors wish to replace the ‘Author Contributions’ statement and the affiliation for Jochen Maurer of this article [1] with the following version since a wrong version was uploaded due to oversight:

Author Contributions: A.M. and F.M. supervised and administered the project. R.A.S., A.M. and F.M. contributed to the study conception and design. R.A.S. and J.P. analyzed and curated the data. A.T.J.V., A.C., S.T. and C.B. conducted chemical syntheses. R.A.S. and J.P. carried out the cell culture, experiments with cell lines, western blot experiments, microscopy and imaging. R.A.S. and M.B. performed statistical analyses. A.T.J.V., C.B., S.T., A.C. and J.M. provided material and resources. R.A.S., J.P., M.B., A.M. and F.M. interpreted the analyzed data. R.A.S. wrote the manuscript. A.M., M.B., F.M. and J.M. corrected the manuscript. All authors have read and agreed to the published version of the manuscript.

Affiliation 5: Department of Gynecology and Obstetrics, University Hospital Aachen, RWTH Aachen University, 52074 Aachen, Germany.

The authors apologize for any inconvenience caused and state that the scientific conclusions are unaffected. The original article has been updated.
