# Supplementary material for: Auger Emitter Conjugated PARP Inhibitor for Therapy in Triple Negative Breast Cancers: A Comparative In-Vitro Study
Source: Cancers (Basel). 2022 Jan 4;14(1):230. doi: 10.3390/cancers14010230 (PMC8750932; doi:10.3390/cancers14010230)
Supplement: Supplementary file 1 [file cancers-14-00230-s001.zip › cancers-1490721 - sup.pdf]

SUPPORTING INFORMATION

for

Auger-emitter conjugated PARP inhibitor for therapy in triple negative breast cancers: a comparative in-vitro study

Ramya Ambur Sankaranarayanan[1], Jennifer Peil[1], Andreas T.J. Vogg[1], Carsten Bolm[2], Steven Terhorst[2], Arno Classen[2], Matthias Bauwens[1,3,4], Jochen Maurer[5], Felix Mottaghy[1,3], Agnieszka Morgenroth[1*].

1. Department of Nuclear Medicine, University Hospital Aachen, RWTH Aachen University, Germany.
2. Institute of Organic Chemistry, RWTH Aachen University, Germany
3. Department of Radiology and Nuclear Medicine, Maastricht University Medical Center (MUMC+), Maastricht, The Netherlands
4. Research School NUTRIM, Maastricht University, Universiteitssingel 50, 6229 ER, Maastricht, The Netherlands.
5. Department of Molecular Gynecology, University Hospital Aachen, RWTH Aachen University, Germany.

*Correspondence: amorgenroth@ukaachen.de.

Table of Content

1. Chemistry – Syntheses and Analyses S2-3
1.1 Synthesis of 4-{3′-[4″-(4‴-iodobenzoyl)piperazine-1″-carbonyl]-4′-fluorobenzyl}-2H-phthalazin-1-one S2
1.2 Synthesis of 4-{3′-[4″-(4‴-tributylstannylbenzoyl)piperazine-1″-carbonyl]-4′-fluorobenzyl}-2H-phthalazin-1-one S2-3
1.3. NMR spectra with supplementary figures S1A-D S4-5
2. Supplementary Figures S6-18
Fig. S2A: Quality control of [^{125}I]-PARPi-01 using gradient Reverse Phase HPLC S6
Fig. S2B: Comparison of ^{125}I vs [^{125}I]-PARPi-01 in TLC S7
1.3. Fig. S3A: Western blot analysis of PARP1 expression S8
1.4. Fig. S3B: Phosphorimager analysis of radioactivity in nuclear lysates S9
1.5. Fig. S4: Immunofluorescence microscopy of PARP1 expression S10
1.6. Fig. S5: Immunofluorescence microscopy of BT20 cells S10
1.7. Fig. S6: Comparison of cell cycle phases S11-13
1.8. Fig. S7: Comparison of early and late apoptotic populations and live cell population S14-16
1.9. Fig. S8: Images of colony formation of cell-lines S17-18

1. Chemistry – Syntheses and Analyses

1.1. Synthesis of 4-{3′-[4″-(4‴-iodobenzoyl)piperazine-1″-carbonyl]-4′-fluorobenzyl}-2H-phthalazin-1-one

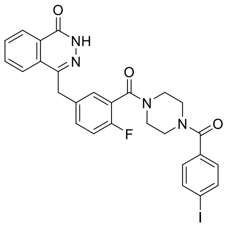

The known compound (F. Zmuda, G. Malviya, A. Blair, M. Boyd, A. J. Chalmers, A. Sutherland, S. L. Pimlott, *J. Med. Chem.* **2015**, *58*, 8683-8693) was prepared by a modified synthetic protocol.

4-[4-Fluoro-3-(piperazine-1-carbonyl)benzyl]phthalazin-1(2H)-one (60 mg, 0.164 mmol, 1 equiv.), 4-iodobenzoic acid (40.7 mg, 0.164 mmol, 1 equiv.), *N*,*N*'-dicyclohexylcarbodiimide (DCC, 37.1 mg, 0.18 mmol, 1.1 equiv.) and 4-dimethylaminopyridine (DMAP, 2 mg, 0.016 mmol, ca. 10 mol%) were used in a typical DCC-coupling reaction with acetonitrile (2 mL) as solvent at room temperature. After completion of the reaction (as indicated by TLC, 16 h), the mixture was diluted with DCM, neutralized with diluted sodium bicarbonate solution and washed with water (3 times). The organic phase was dried over magnesium sulfate and concentrated under reduced pressure. The product was purified by column chromatography (EtOAc 100%) and obtained as a colorless powder in 85% yield (84.2 mg).

^{1}H NMR (600 MHz, Chloroform-*d*) δ 11.57 (s, 1H), 8.59 – 8.23 (m, 1H), 7.82 – 7.67 (m, 5H), 7.33 (d, *J* = 6.0 Hz, 2H), 7.13 (d, *J* = 6.1 Hz, 2H), 7.02 (s, 1H), 4.28 (s, 2H), 3.83 – 3.20 (m, 8H). ^{13}C NMR (151 MHz, Chloroform-*d*) δ 169.7, 165.2, 161.0, 156.9 (d, *J* = 247.4 Hz), 145.6, 137.8, 134.5 (d, *J* = 3.2 Hz), 134.4, 133.7, 131.8 (d, *J* = 7.8 Hz), 131.6, 129.5, 129.3 (d, *J* = 3.6 Hz), 128.8, 128.2, 127.2, 125.0, 123.5 (d, *J* = 17.8 Hz), 116.2 (d, *J* = 21.8 Hz), 96.5, 47.3, 42.2, 37.7.

1.2. Synthesis of 4-{3′-[4″-(4‴-tributylstannylbenzoyl)piperazine-1″-carbonyl]-4′-fluorobenzyl}-2H-phthalazin-1-one

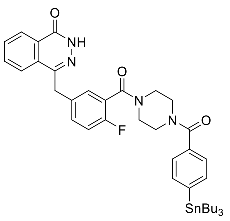

A protocol for the synthesis of an isomeric product (as reported in t. C. Wilson, S. A. Jannetii, N. Guru, N. Pillarsetty, T. Reiner, G. Pirovano, *Int. J. Radiation Biol.*, DOI 10.1080/09553002.2020.1781283) was adopted and modified.

To a solution of 4-[4-fluoro-3-(piperazine-1-carbonyl)benzyl]phthalazin-1(2H)-one (30 mg, 0.0819 mmol, 1 equiv.) and 2-(1*H*-benzotriazol-1-yl)-1,1,3,3-tetramethyluronium hexafluorophosphate (HBTU, 48 mg, 0.123 mmol, 1.5 equiv.) in acetonitrile (1.5 mL), 1-[4-(tributylstannyl)benzoyl]-pyrrolidine-2,5-dione (41.6 mg, 0.0819 mmol, 1 equiv.) was added at 30 °C. After stirring for 3 h at 30 °C and 16 h at room temperature, the mixture was diluted with water and washed with DCM (3 times). The combined organic phases were dried over magnesium sulfate and concentrated under reduced

pressure. The product was purified by column chromatography (ethyl acetate/cyclohexane 5:1) and obtained as an oil in 39% yield (24.2 mg).

^{1}H NMR (600 MHz, Chloroform-d) δ 11.08 (s, 1H), 8.46 (dd, *J* = 6.9, 2.2 Hz, 1H), 7.96 – 7.66 (m, 3H), 7.50 (s, 2H), 7.40 – 7.27 (m, 4H), 7.03 (s, 1H), 4.29 (s, 2H), 3.87 – 3.19 (m, 8H), 1.52 (t, *J* = 8.1 Hz, 6H), 1.32 (q, *J* = 7.4 Hz, 6H), 1.05 (t, *J* = 8.2 Hz, 6H), 0.87 (t, *J* = 7.3 Hz, 9H). ^{13}C NMR (151 MHz, Chloroform-d) δ 171.1, 165.3, 160.8, 157.1 (d, *J* = 248.3 Hz), 145.7, 136.7, 134.5 (d, *J* = 7.9 Hz), 133.9, 131.8, 129.7, 129.4 (d, *J* = 3.6 Hz), 128.4, 127.3, 126.3, 125.2, 123.8 (d, *J* = 18.7 Hz), 116.3 (d, *J* = 22.4 Hz), 47.3, 42.4, 37.8, 29.1, 27.5, 13.8, 9.8. [$C_{39}H_{49}FN_4O_3SnNa^+$] Mass (calc): 783.2703; Found: 783.2662.

1.3. NMR spectra

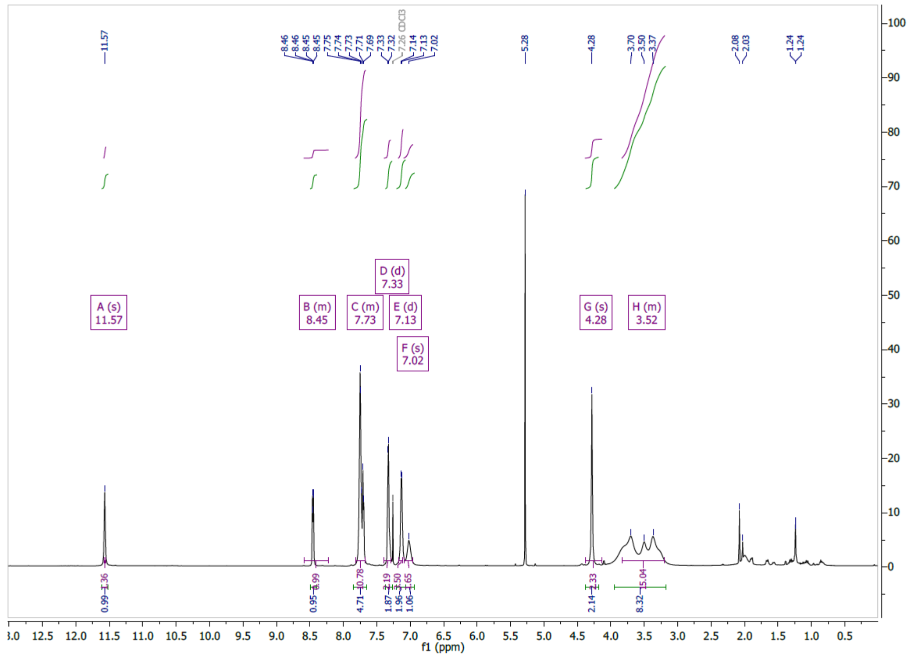

Fig. S1A: ^{1}H NMR spectrum of 4-{3′-[4′′-(4′′′-iodobenzoyl)piperazine-1′′-carbonyl]-4′-fluorobenzyl}-2H-phthalazin-1-one

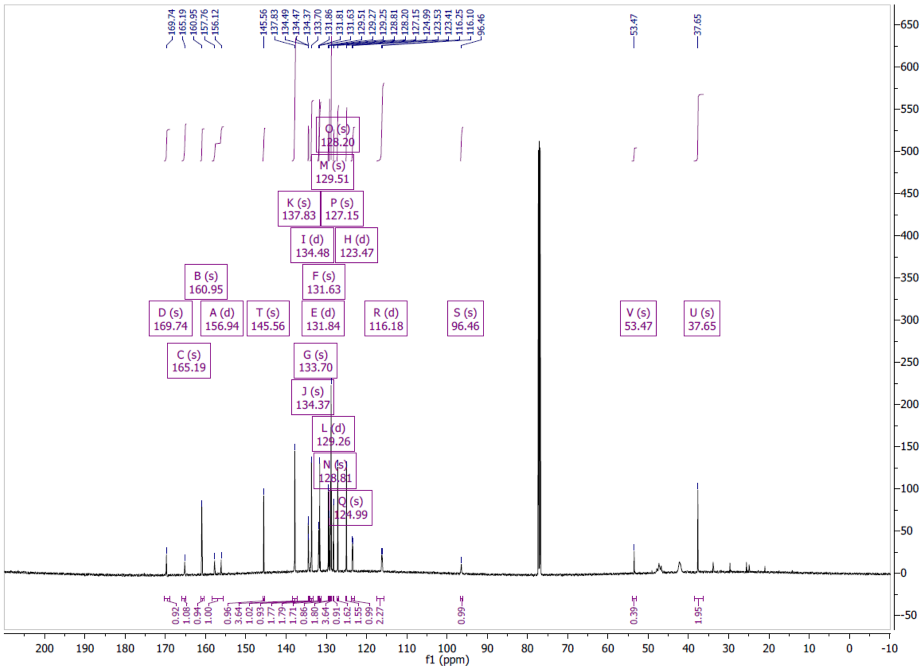

Fig. S1B: ^{13}C{^{1}H} NMR spectrum of 4-{3′-[4′′-(4′′′-iodobenzoyl)piperazine-1′′-carbonyl]-4′-fluorobenzyl}-2H-phthalazin-1-one

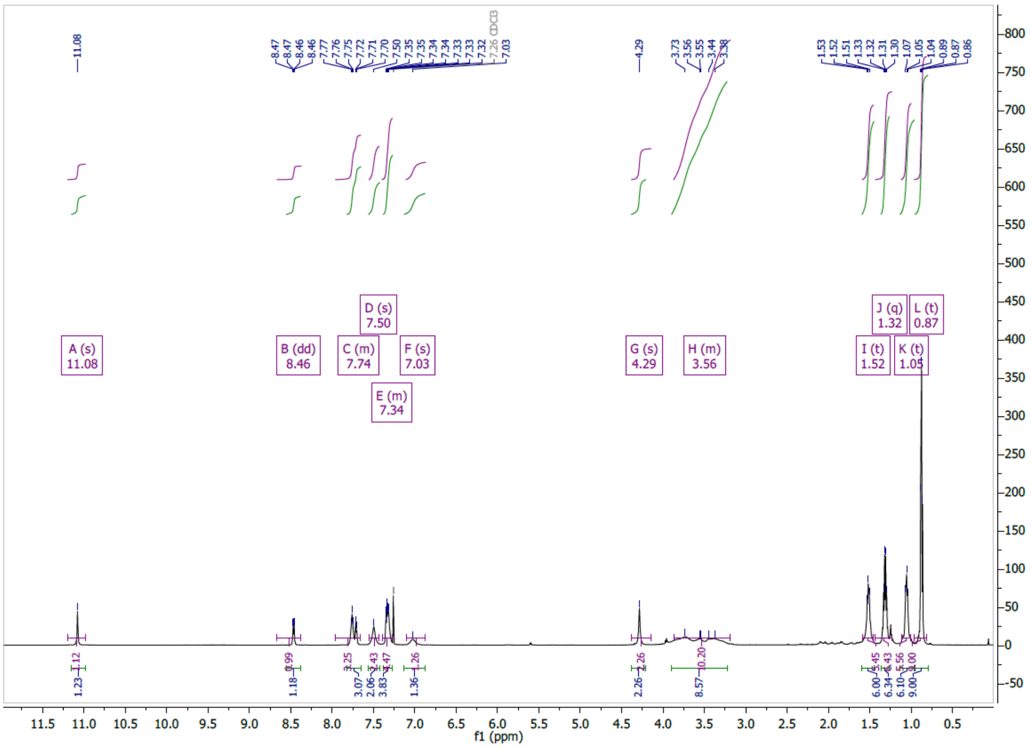

Fig. S1C: ^{1}H NMR spectrum of 4-{3′-[4′′-(4′′′-tributylstannylbenzoyl)piperazine-1′′-carbonyl]-4′-fluorobenzyl}-2H-phthalazin-1-one

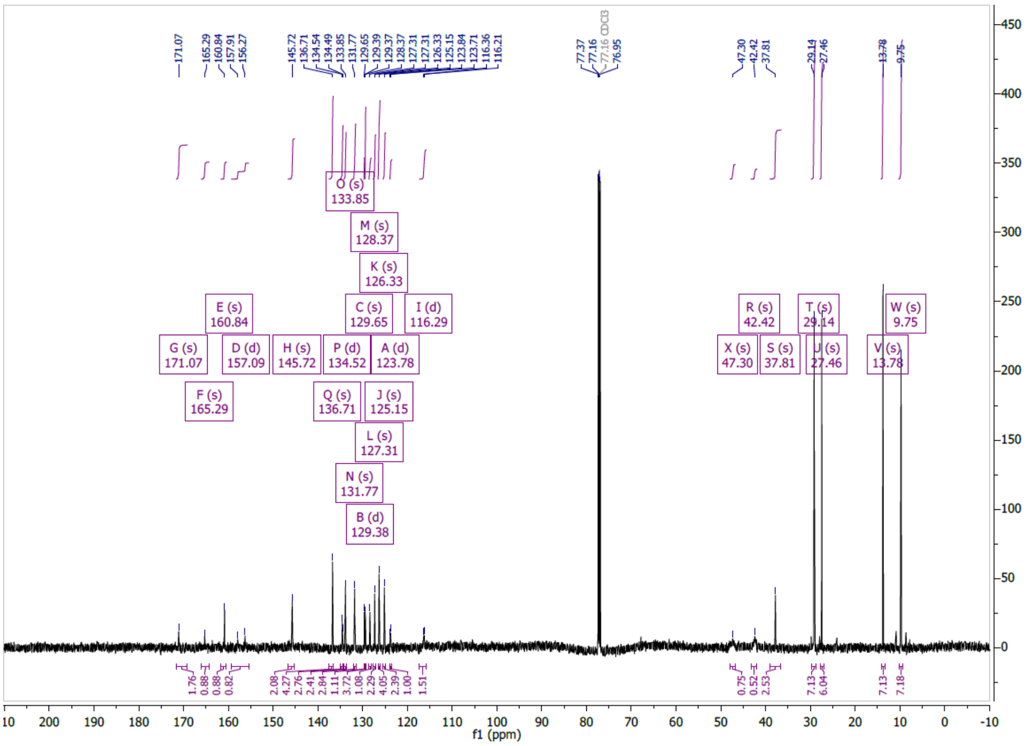

Fig. S1D: ^{13}C{^{1}H} NMR spectrum of 4-{3′-[4′′-(4′′′-tributylstannylbenzoyl)piperazine-1′′-carbonyl]-4′-fluorobenzyl}-2H-phthalazin-1-one

2. Supplementary Figures

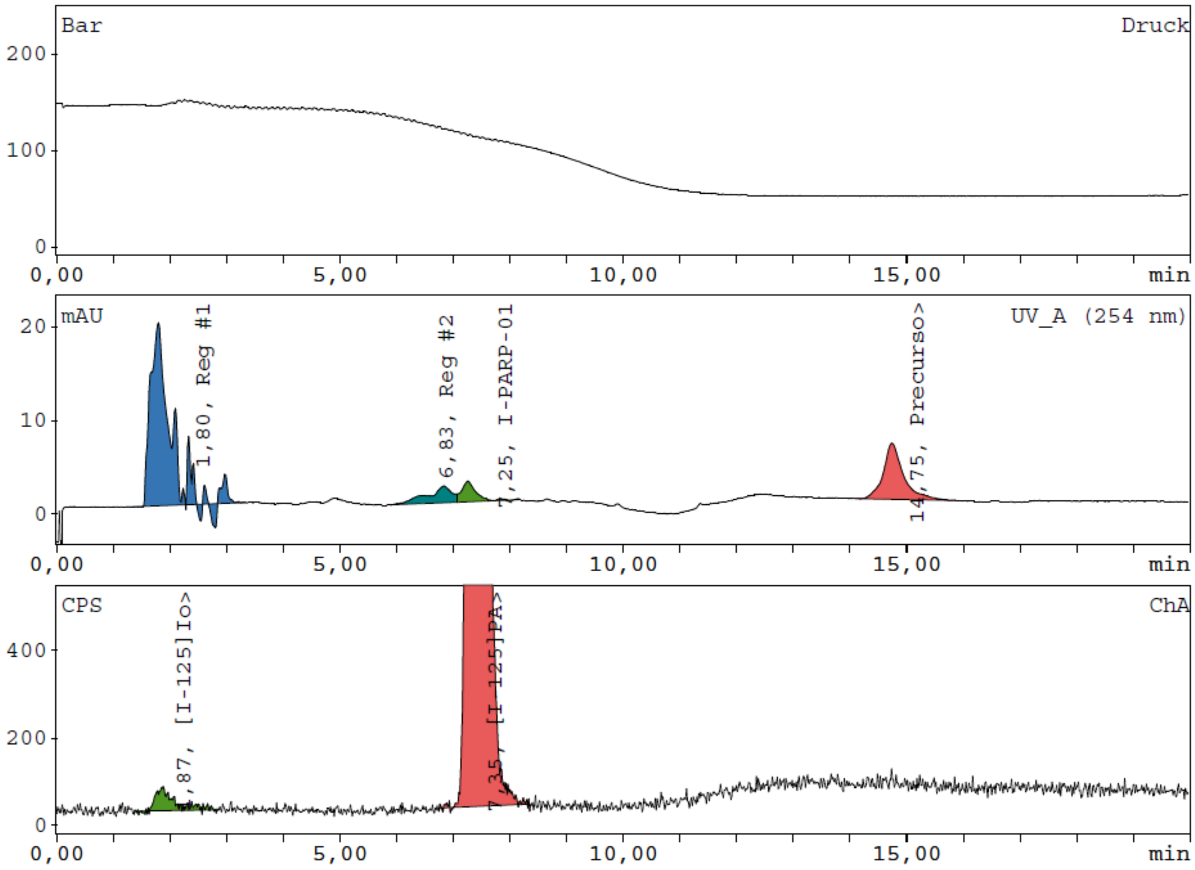

Fig. S2A: Quality control of [^{125}I]-PARPi-01 using gradient Reverse Phase HPLC. HPLC system contains UV and gamma detection. **HPLC conditions**: LiChrospher 100 RP18 EC 5µm, 250x4 mm, Flow 1 mL/min, pressure ca. 140 bar, eluents: A: CH_3CN, B: H_2O (0.1% TFA, citrate), gradient: 0-2 min 40% A / 2-8 min 40% A -> 100% A / 8-18 min 100% A / 18-20 min 40% A. UV absorbance: 254 nm. Retention times: [^{125}I]iodide (2 min), [^{125}I]PARPi-01 (7.5 min), labelling precursor (14.8 min). Radiochemical product purity in this example (20.08.2021) is 99.2 %.

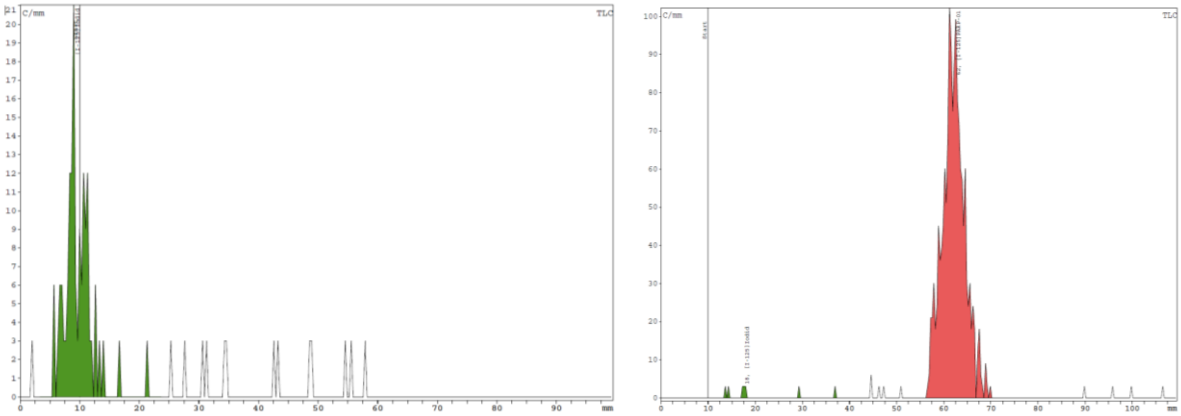

Fig. S2B: Comparison of ^{125}I (green peak) vs [^{125}I]-PARPi-01 (red peak) in thin layer chromatography (TLC) followed by scanning using a radio-TLC scanner as quality control 3 months post synthesis upon storage at –20°C (Solvent: Ethanol). No deiodination observed upon long term storage. **TLC run conditions**: Mobile phase: Acetone; Stationary phase: Silica on Aluminium backing.

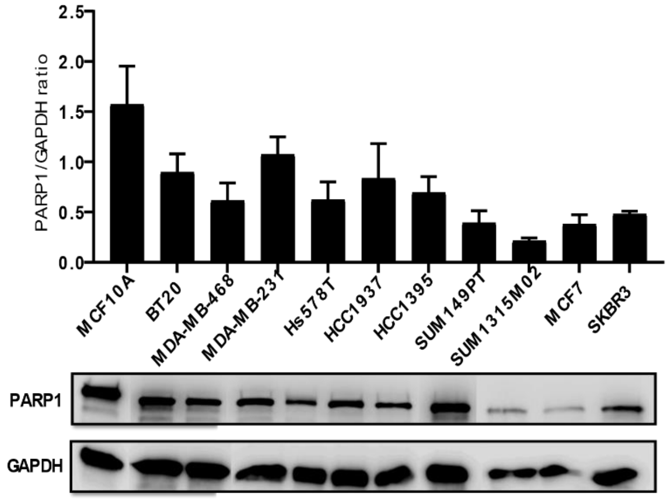

Fig. S3A: Western blot analysis of PARP1 expression in mammary epithelial control (MCF10A) and TNBC and breast cancer cell lines.

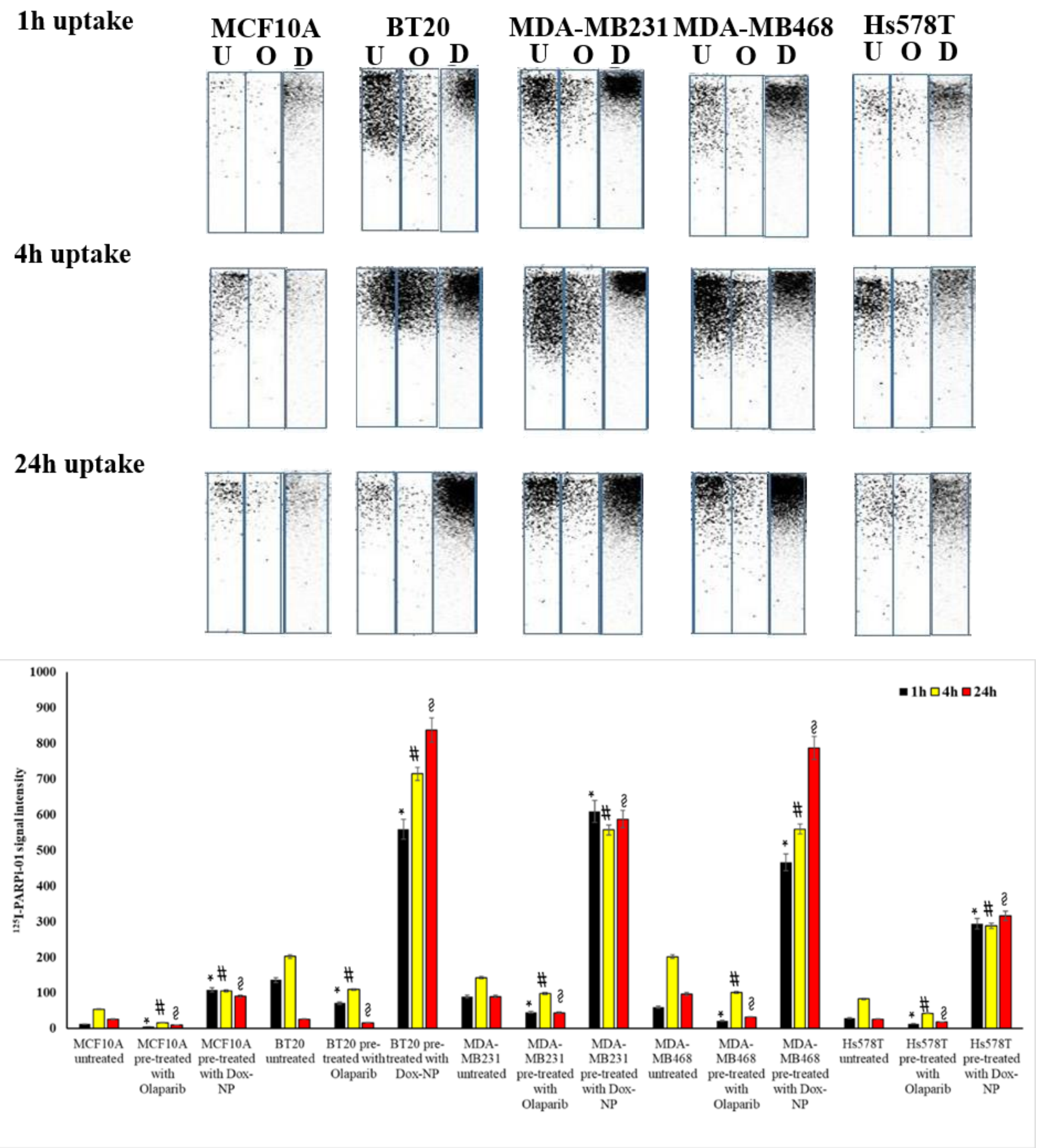

Fig S3B: Phosphor-imager (PI) analysis of radioactivity in nuclear lysates of representative cell lines run in SDS-PAGE. Untreated (U – above PI picture) cells were incubated with $[^{125}I]$-PARPi-01 without pre-treatment; Olaparib pre-treated (O – above PI picture) cells were treated with 1µM Olaparib for 72hrs; Dox-NP (D – above PI picture) cells were pre-treated with 100nM Dox-NP for 6hrs. After incubation with $[^{125}I]$-PARPi-01 (1MBq/10^6 cells) for 1hr, 4hrs and 24hrs cells were harvested, fractionated and nuclear lysates were run in SDS-PAGE. The radioactivity was detected and visualised using Phosphor-imager. As opposed to the control MCF10A (benign mammary epithelial) the breast cancer cell lines show enhanced uptake upon Dox-NP pre-treatment. Quantification of the signal intensity was performed using Image Quant TL Toolbox v8.1. Statistical analysis performed using 2-way ANOVA using Dunnetts post-hoc test. *P<0.001 at 1h; $^{\#}$ P<0.001 at 4h; §P<0.001 at 24h. Significance test was performed within each cell-line.

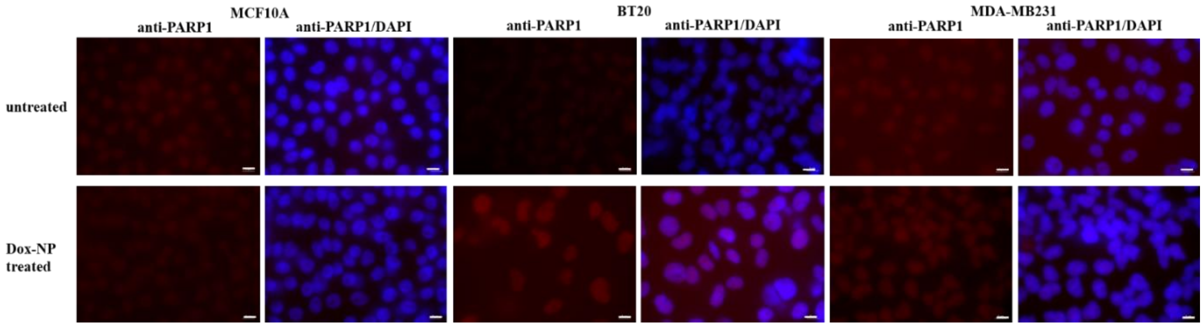

Fig. S4: Immunofluorescence microscopy of PARP1 expression in untreated and DOX-NP (100 nM for 6 hrs) treated benign and malignant breast cells. Scale bars represent 5µm.

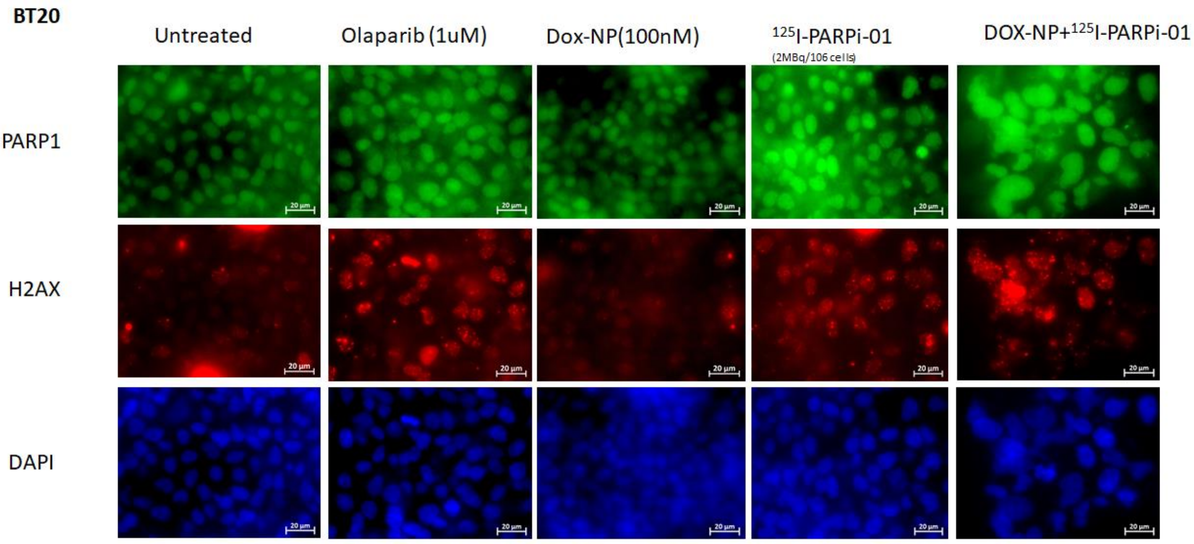

Fig. S5: Representative Immunofluorescence images of BT20 cell-line treated with Olaparib (72 hrs), Dox-NP (6 hrs) or [^{125}I]-PARPi-01 (2MBq/10^6 cells,72hrs) or combination of Dox-NP pretreatment followed by [^{125}I]-PARPi-01 and stained for PARP1 expression levels (green), H2AX foci (red) along with DAPI (nucleus).

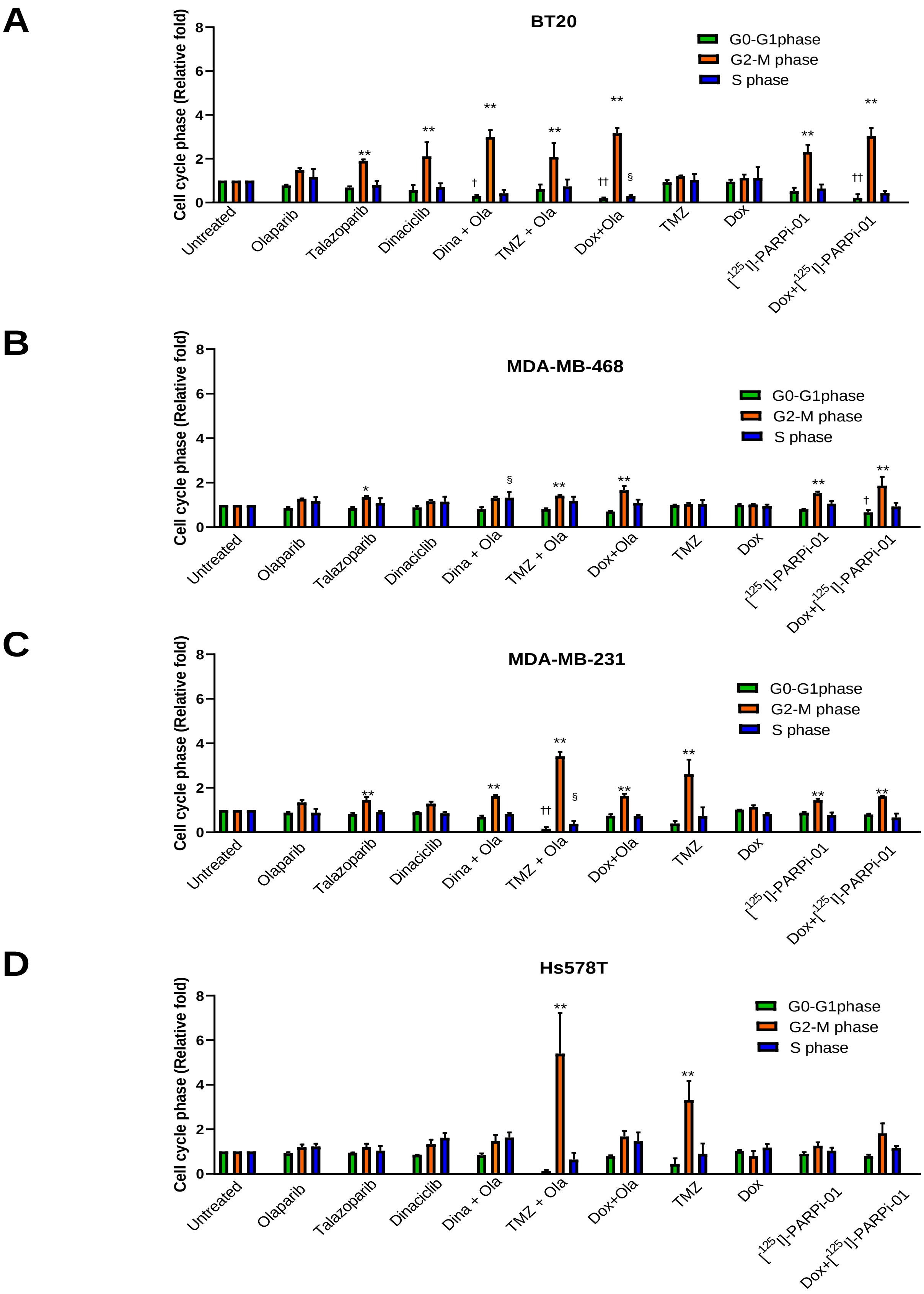
A
BT20
Cell cycle phase (Relative fold)
0
2
4
6
8
G0-G1phase
G2-M phase
S phase
Untreated
Olaparib
Talazoparib
Dinaciclib
Dina + Ola
TMZ + Ola
Dox+Ola
TMZ
Dox
[125I]-PARPi-01
Dox+[125I]-PARPi-01
B
MDA-MB-468
Cell cycle phase (Relative fold)
0
2
4
6
8
G0-G1phase
G2-M phase
S phase
Untreated
Olaparib
Talazoparib
Dinaciclib
Dina + Ola
TMZ + Ola
Dox+Ola
TMZ
Dox
[125I]-PARPi-01
Dox+[125I]-PARPi-01
C
MDA-MB-231
Cell cycle phase (Relative fold)
0
2
4
6
8
G0-G1phase
G2-M phase
S phase
Untreated
Olaparib
Talazoparib
Dinaciclib
Dina + Ola
TMZ + Ola
Dox+Ola
TMZ
Dox
[125I]-PARPi-01
Dox+[125I]-PARPi-01
D
Hs578T
Cell cycle phase (Relative fold)
0
2
4
6
8
G0-G1phase
G2-M phase
S phase
Untreated
Olaparib
Talazoparib
Dinaciclib
Dina + Ola
TMZ + Ola
Dox+Ola
TMZ
Dox
[125I]-PARPi-01
Dox+[125I]-PARPi-01

E

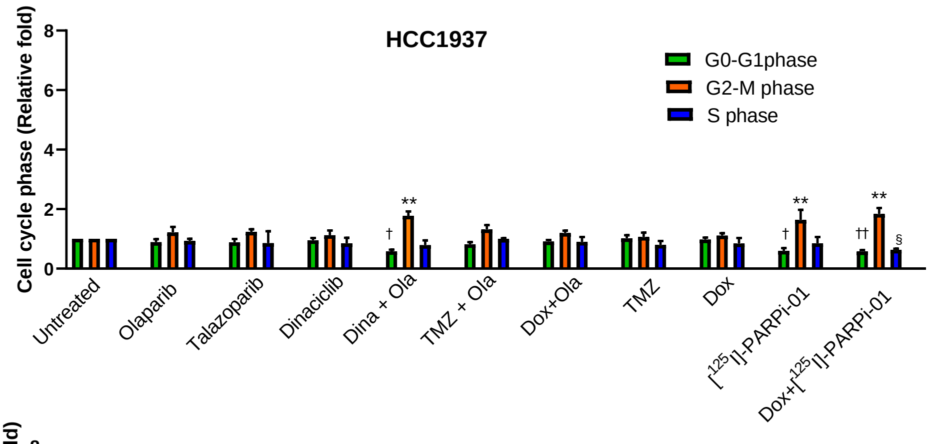
HCC1937
Cell cycle phase (Relative fold)
0
2
4
6
8
G0-G1phase
G2-M phase
S phase
†
**
†
**
††
**
§
Untreated
Olaparib
Talazoparib
Dinaciclib
Dina + Ola
TMZ + Ola
Dox+Ola
TMZ
Dox
[125I]-PARPi-01
Dox+[125I]-PARPi-01

F

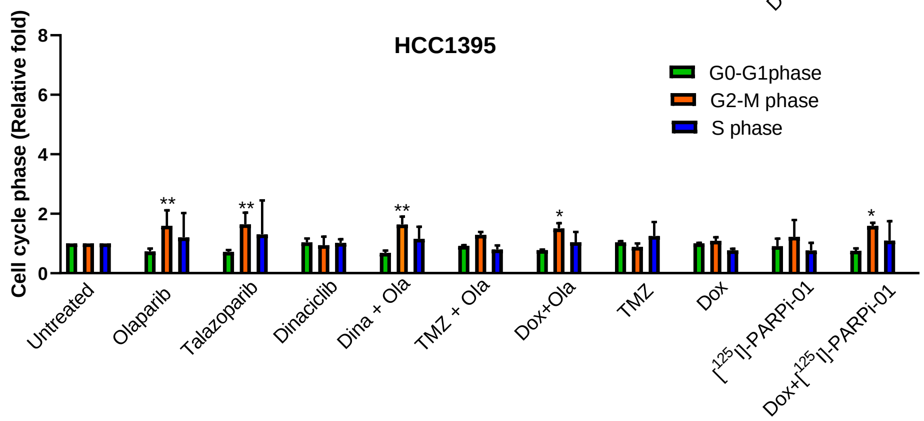
HCC1395
Cell cycle phase (Relative fold)
0
2
4
6
8
G0-G1phase
G2-M phase
S phase
**
**
**
*
*
Untreated
Olaparib
Talazoparib
Dinaciclib
Dina + Ola
TMZ + Ola
Dox+Ola
TMZ
Dox
[125I]-PARPi-01
Dox+[125I]-PARPi-01

G

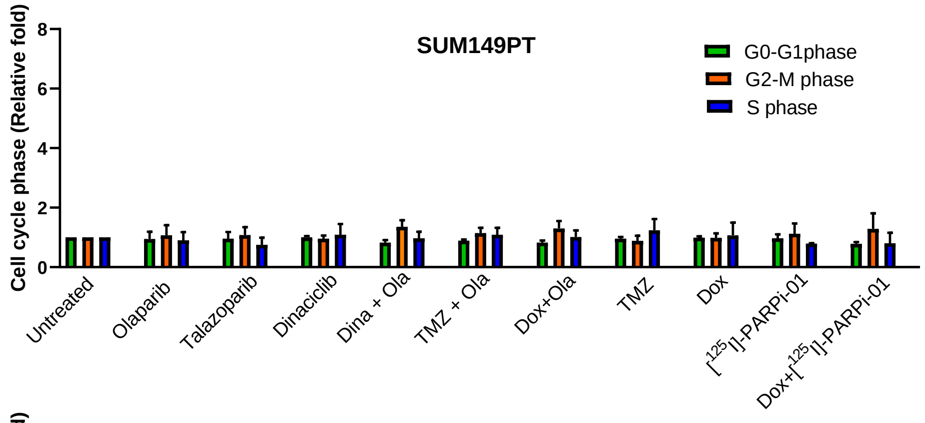
SUM149PT
Cell cycle phase (Relative fold)
0
2
4
6
8
G0-G1phase
G2-M phase
S phase
Untreated
Olaparib
Talazoparib
Dinaciclib
Dina + Ola
TMZ + Ola
Dox+Ola
TMZ
Dox
[125I]-PARPi-01
Dox+[125I]-PARPi-01

H

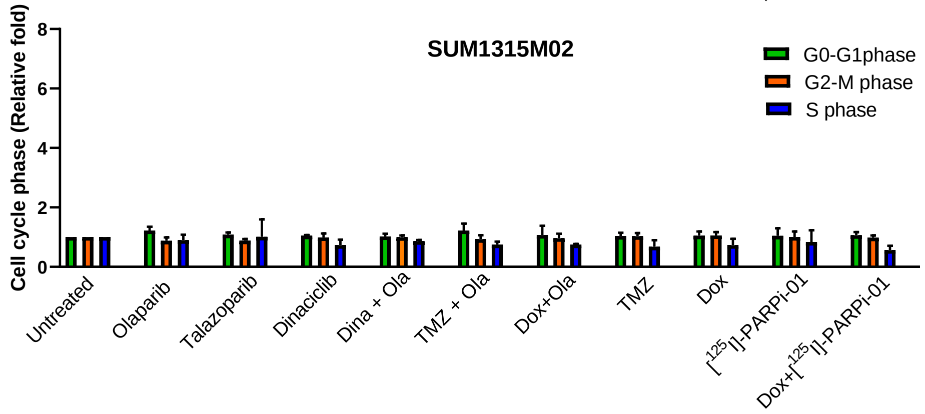
SUM1315M02
Cell cycle phase (Relative fold)
0
2
4
6
8
G0-G1phase
G2-M phase
S phase
Untreated
Olaparib
Talazoparib
Dinaciclib
Dina + Ola
TMZ + Ola
Dox+Ola
TMZ
Dox
[125I]-PARPi-01
Dox+[125I]-PARPi-01

I

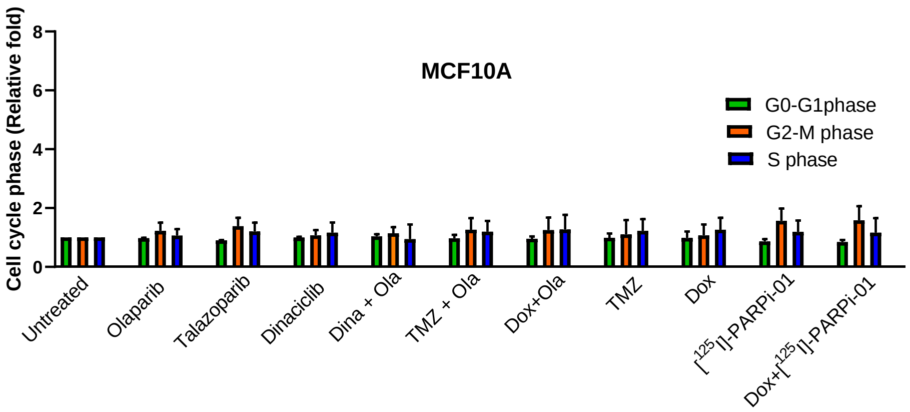

J

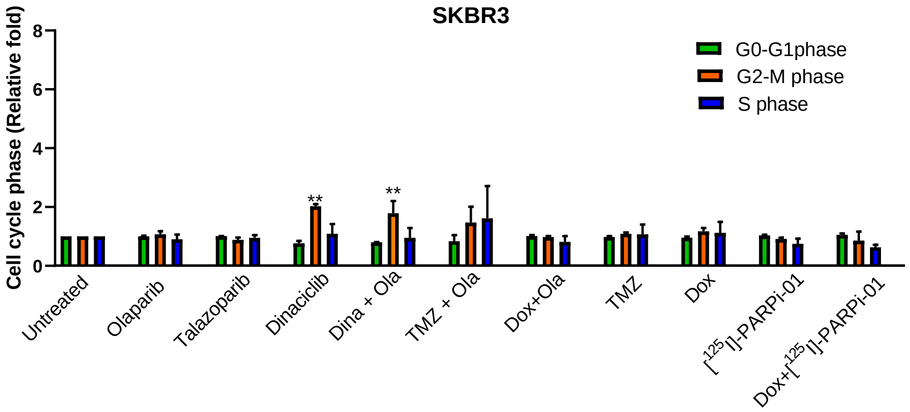

K

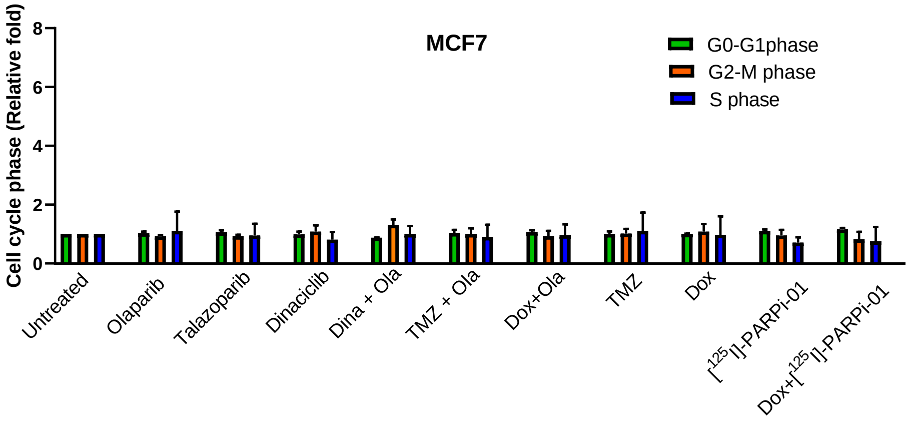

Fig. S6: Comparison of cell cycle phases upon treatments with olaparib (ola, 1 µM, 72 hrs), talazoparib (tala, 10 nM, 72 hrs), Dinaciclib (Dina, 5 µM, 72 hrs), temozolomide (TMZ, 50 µM, 48 hrs), Dox-NP (Dox, 100 nM, 6 hrs), [^{125}I]-PARP-01 and combination treatments: Dina (5 nM)+Ola (1 µM) (72 hrs), TMZ (48 hrs)+Ola (24 hrs), Dox-NP (6 hrs)+Ola (60 hrs) in each cell line **A**. BT20. **B.** MDA-MB-468 **C**. MDA-MB-231, **D.** Hs578T; **E**. HCC1937; **F**. HCC1395; **G**. SUM149PT; **H**. SUM1315M02; **I**. MCF10A; **J.** SKBR3; **K**. MCF7. ; Statistical analysis was calculated using 2-way ANOVA and Bonferroni post-hoc test. * indicates signicances between G2-M phase, † indicates significance between G0-G1 phase and § indicates significance between S phase. (*P<0.05; **P<0.001; †P<0.05; ††P<0.001; §P<0.05).

A

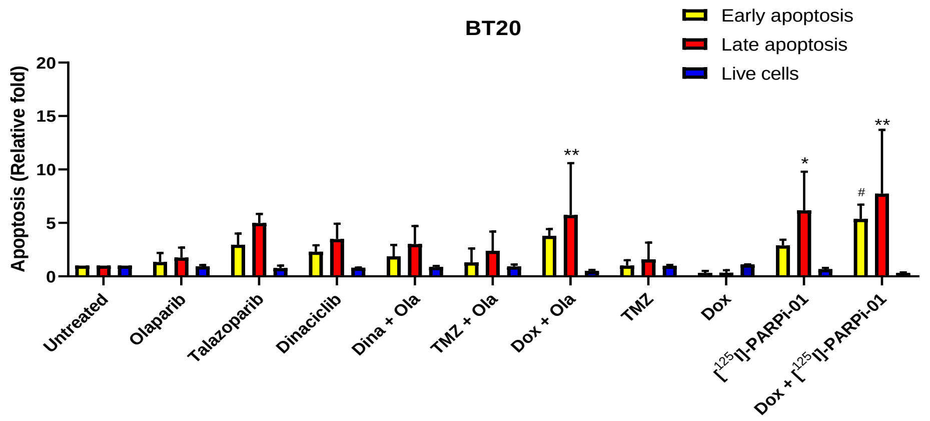
BT20
Early apoptosis
Late apoptosis
Live cells
Apoptosis (Relative fold)
0
5
10
15
20
**
*
#
**
Untreated
Olaparib
Talazoparib
Dinaciclib
Dina + Ola
TMZ + Ola
Dox + Ola
TMZ
Dox
[125I]-PARPi-01
Dox + [125I]-PARPi-01

B

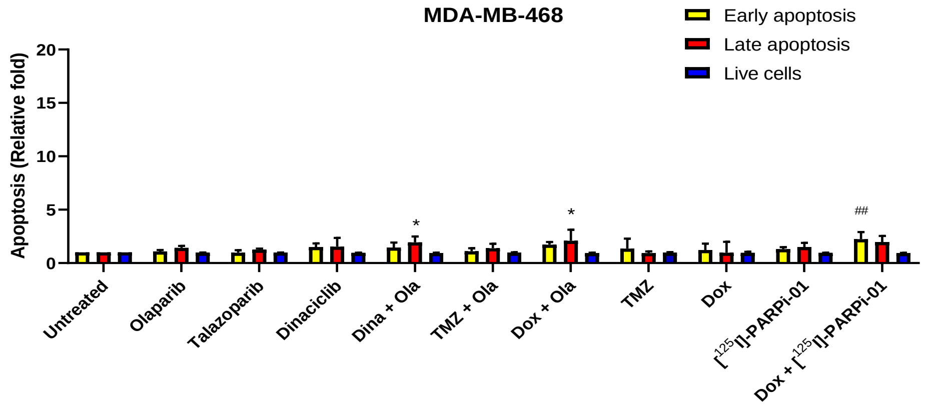
MDA-MB-468
Early apoptosis
Late apoptosis
Live cells
Apoptosis (Relative fold)
0
5
10
15
20
*
*
##
Untreated
Olaparib
Talazoparib
Dinaciclib
Dina + Ola
TMZ + Ola
Dox + Ola
TMZ
Dox
[125I]-PARPi-01
Dox + [125I]-PARPi-01

C

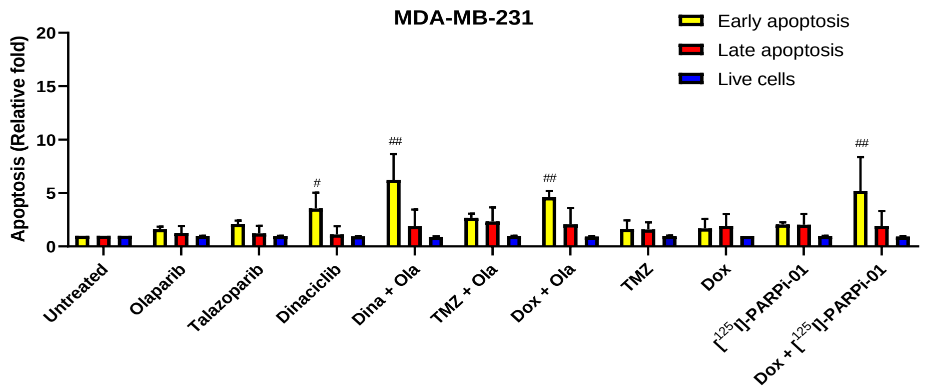
MDA-MB-231
Early apoptosis
Late apoptosis
Live cells
Apoptosis (Relative fold)
0
5
10
15
20
#
##
##
##
Untreated
Olaparib
Talazoparib
Dinaciclib
Dina + Ola
TMZ + Ola
Dox + Ola
TMZ
Dox
[125I]-PARPi-01
Dox + [125I]-PARPi-01

D

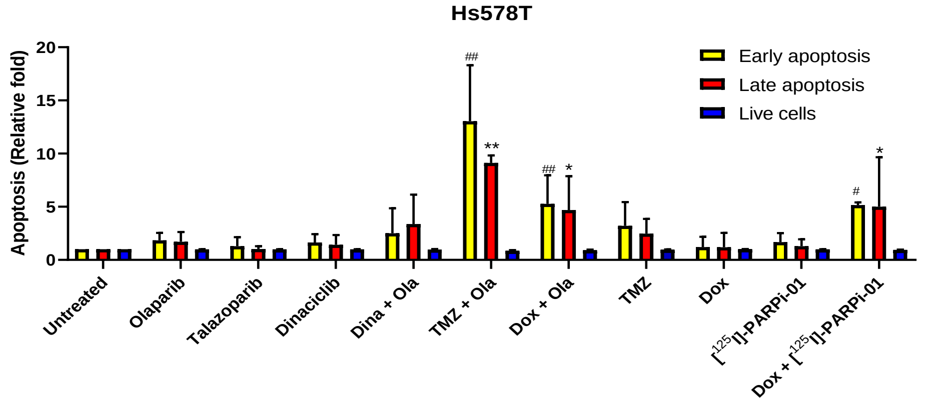
Hs578T
Early apoptosis
Late apoptosis
Live cells
Apoptosis (Relative fold)
0
5
10
15
20
##
**
##
*
#
*
Untreated
Olaparib
Talazoparib
Dinaciclib
Dina + Ola
TMZ + Ola
Dox + Ola
TMZ
Dox
[125I]-PARPi-01
Dox + [125I]-PARPi-01

E

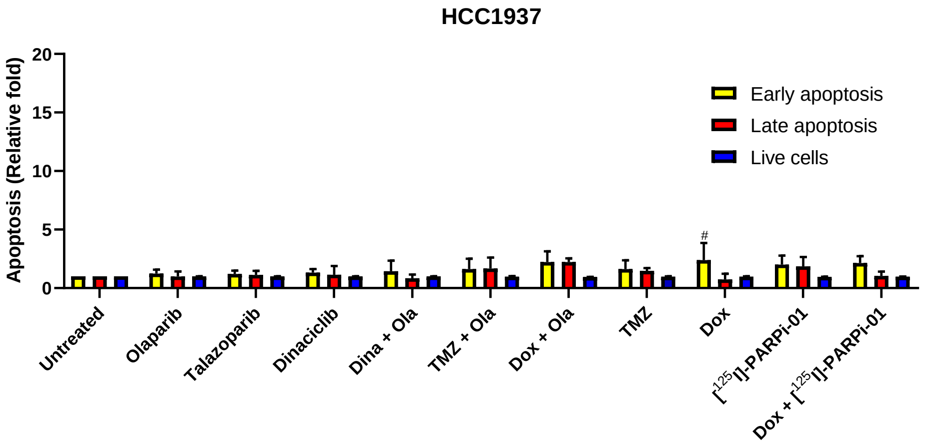
HCC1937
Apoptosis (Relative fold)
0
5
10
15
20
Early apoptosis
Late apoptosis
Live cells
#
Untreated
Olaparib
Talazoparib
Dinaciclib
Dina + Ola
TMZ + Ola
Dox + Ola
TMZ
Dox
[125I]-PARPi-01
Dox + [125I]-PARPi-01

F

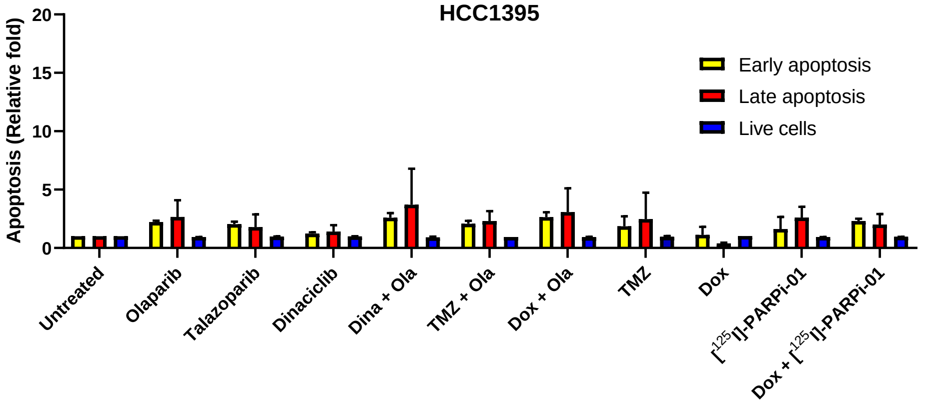
HCC1395
Apoptosis (Relative fold)
0
5
10
15
20
Early apoptosis
Late apoptosis
Live cells
Untreated
Olaparib
Talazoparib
Dinaciclib
Dina + Ola
TMZ + Ola
Dox + Ola
TMZ
Dox
[125I]-PARPi-01
Dox + [125I]-PARPi-01

G

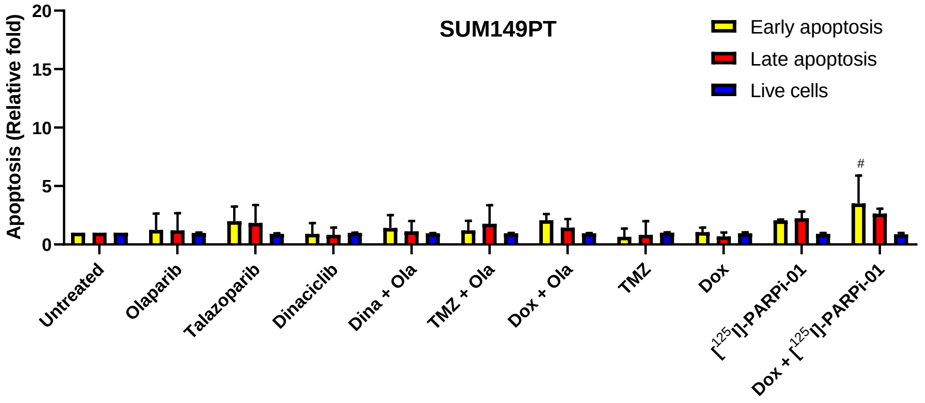
SUM149PT
Apoptosis (Relative fold)
0
5
10
15
20
Early apoptosis
Late apoptosis
Live cells
#
Untreated
Olaparib
Talazoparib
Dinaciclib
Dina + Ola
TMZ + Ola
Dox + Ola
TMZ
Dox
[125I]-PARPi-01
Dox + [125I]-PARPi-01

H

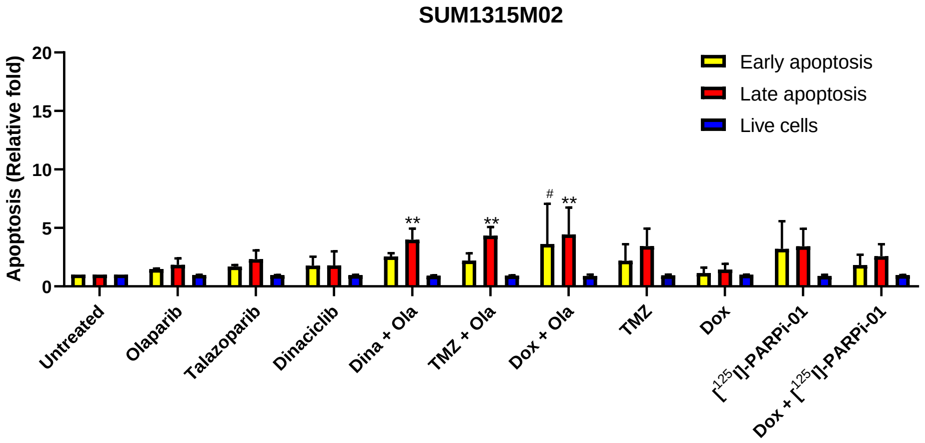
SUM1315M02
Apoptosis (Relative fold)
0
5
10
15
20
Early apoptosis
Late apoptosis
Live cells
**
**
#
**
Untreated
Olaparib
Talazoparib
Dinaciclib
Dina + Ola
TMZ + Ola
Dox + Ola
TMZ
Dox
[125I]-PARPi-01
Dox + [125I]-PARPi-01

I

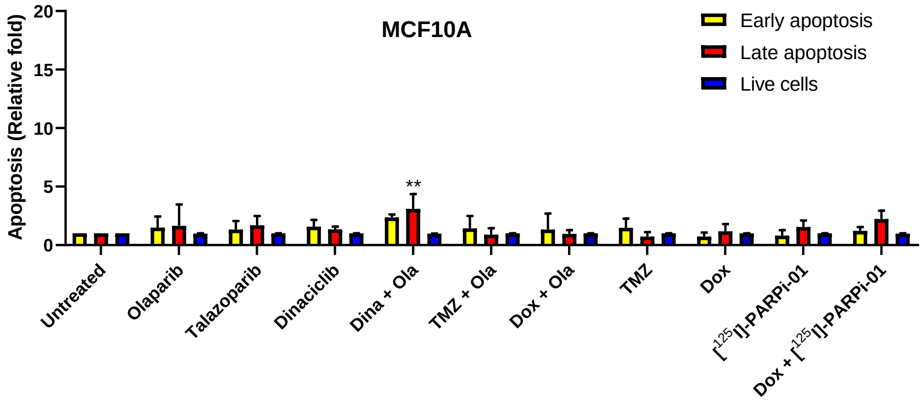

J

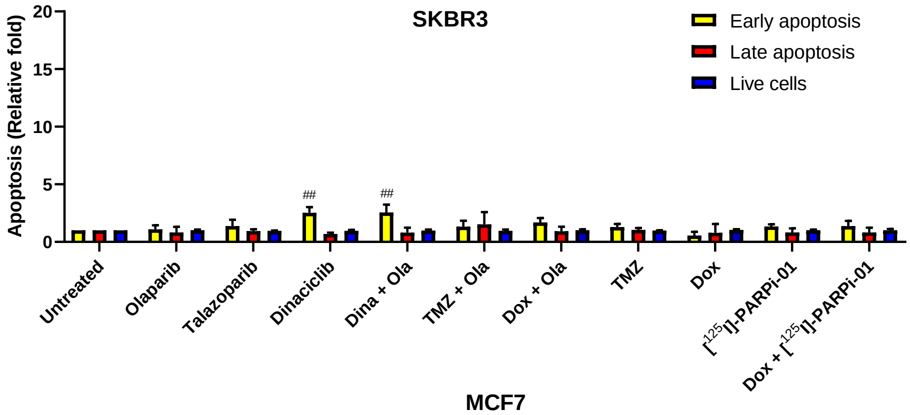

K

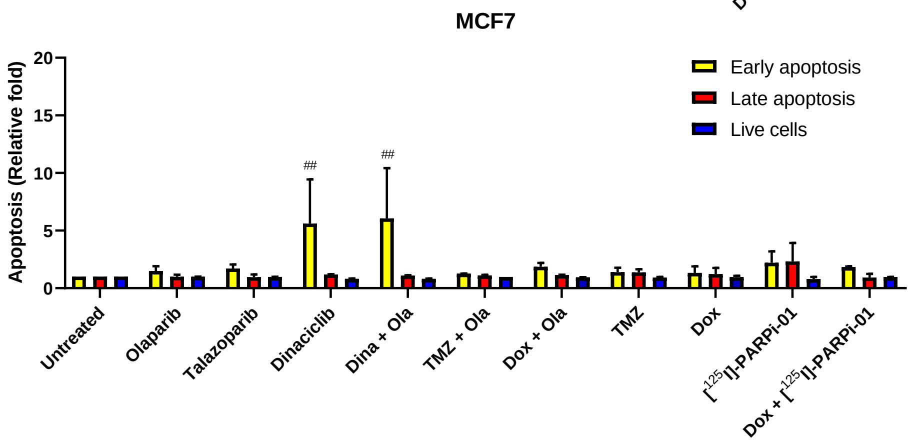

Fig. S7: Comparison of early and late apoptotic populations and live cell population upon treatments with olaparib (ola, 1 µM, 72 hrs), talazoparib (tala, 10nM, 72 hrs), Dinaciclib (Dina, 5 µM, 72 hrs), temozolomide (TMZ, 50 µM, 48hrs), Dox-NP (Dox, 100 nM, 6 hrs). $[^{125}I]$-PARP-01 and combination treatments: Dina (5nM)+Ola (1µM) (72 hrs), TMZ (48 hrs)+Ola(24 hrs), Dox-NP(6 hrs)+Ola(60 hrs) in each cell line **A**. BT20. **B.** MDA-MB-468 **C**. MDA-MB-231, **D.** Hs578T; **E**. HCC1937; **F**. HCC1395; **G**. SUM149PT; **H**. SUM1315M02; **I**. MCF10A; **J.** SKBR3; **K**. MCF7. Statistical analysis was calculated using 2-way ANOVA and Bonferroni post-hoc test. * indicates signicances between Late apoptosis, $^{\#}$ indicates significance between Early apoptosis. (*P<0.05; **P<0.001; $^{\#}$P<0.05; $^{\#\#}$P<0.001).

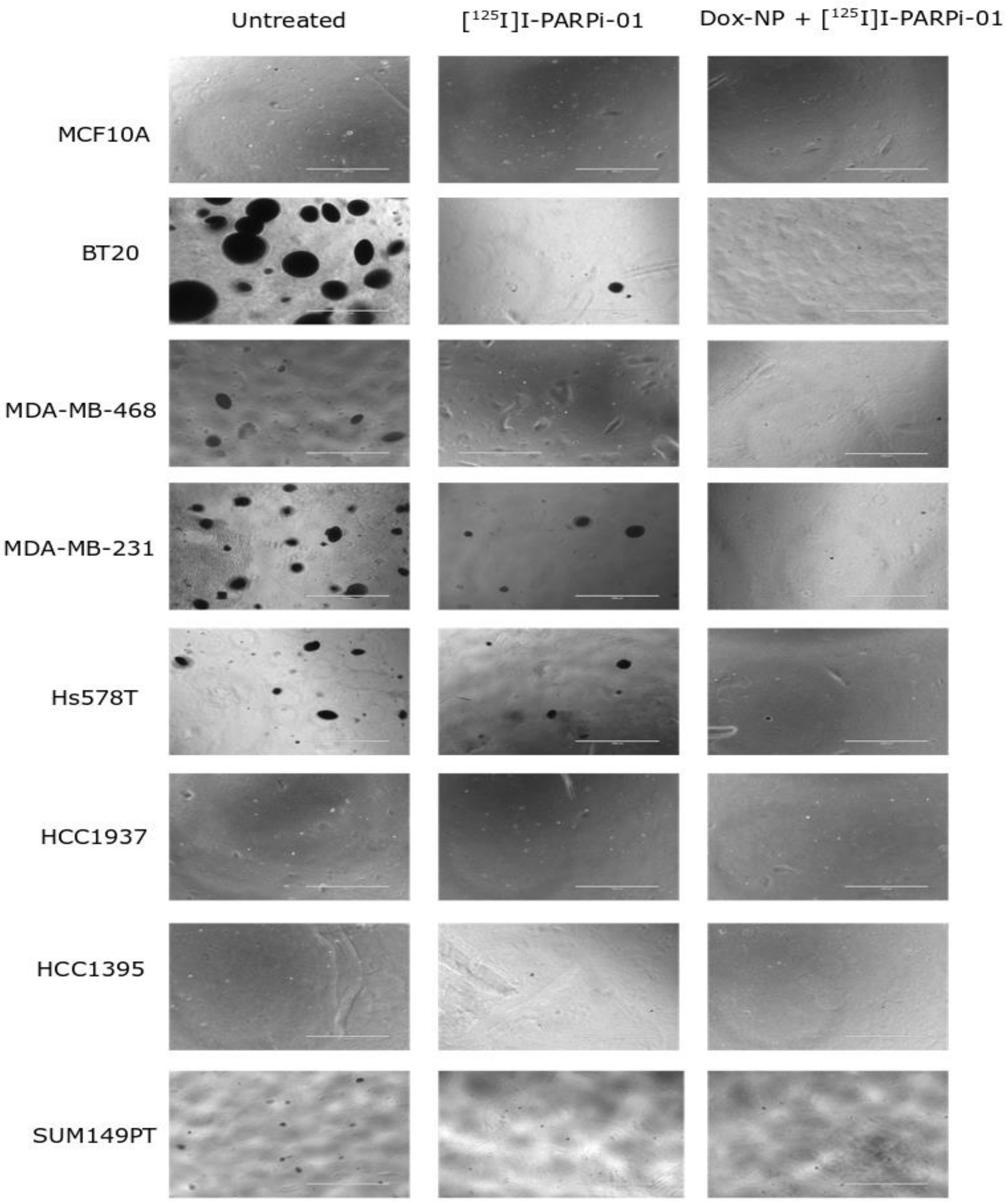
Untreated
[125I]I-PARPi-01
Dox-NP + [125I]I-PARPi-01
MCF10A
BT20
MDA-MB-468
MDA-MB-231
Hs578T
HCC1937
HCC1395
SUM149PT

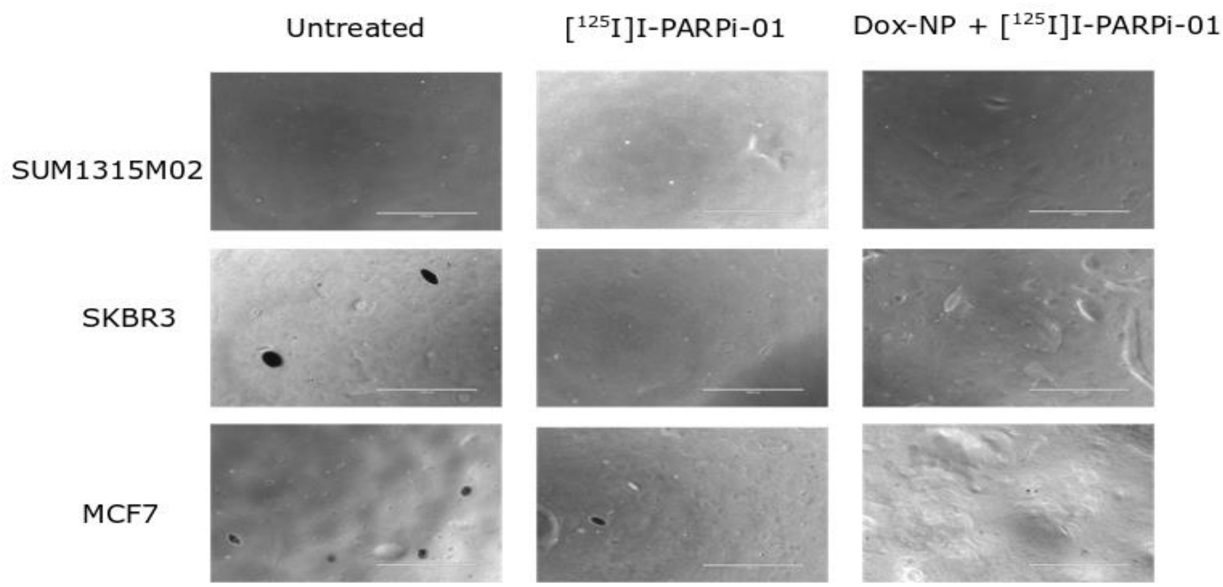

Fig. S8: Images of colony formation of cell-lines upon growth in soft-agar for 21 days post treatment with [^{125}I]-PARPi-01 (2MBq/10^{6} cells, 72 hrs) or combination of Dox-NP pre-treatment (100nM, 6 hrs) followed by [^{125}I]-PARPi-01 (2MBq/10^{6} cells, 72 hrs).
